# Inflammatory Bowel Disease Mediates the Causal Relationship Between Gut Microbiota and Colorectal Cancer: Identification of Therapeutic Targets and Predictive Modeling

**DOI:** 10.7150/jca.114687

**Published:** 2025-09-22

**Authors:** Jin-Bei Wang, Zhen-Guo Wu, Guan-Wei Bi, Yu Li, Zhi-Wen Yao, Yan-Bo Yu

**Affiliations:** 1 First Clinical College, Shandong University, Jinan, Shandong Province, PR China.; 2 Department of Gastroenterology, Qilu Hospital, Shandong University, Jinan, Shandong, PR China.; 3 Shandong Provincial Clinical Research Center for Digestive Disease, Qilu Hospital, Shandong University, Jinan, Shandong Province, PR China.

**Keywords:** colorectal cancer, gut microbiota, inflammatory bowel disease, machine learning, mendelian randomization, transcriptome analysis

## Abstract

**Background:** Colorectal cancer (CRC) is the second leading cause of cancer-related mortality. Given its established associations with gut microbiota and inflammatory bowel disease (IBD), elucidating their relationships and developing predictive models are critical for early detection and therapy.

**Methods:** Using Mendelian randomization (MR), we integrated data from the MiBioGen Consortium and multiple genome-wide association studies (GWAS). Single nucleotide polymorphisms (SNPs) associated with gut microbiota were mapped to genes, followed by gene selection via least absolute shrinkage and selection operator (LASSO) regression. Transcriptome analyses identified differential gene expressions and immune cell infiltration patterns. Six machine learning models were integrated through soft voting to predict CRC risk, validated by single-cell sequencing analysis.

**Results:** Mediation MR identified 12 gut microbial taxa causally associated with CRC, mediated partially by IBD. SNP mapping and expression analysis highlighted eight CRC-associated genes, five of which (FAM120A, GBE1, MCM6, MSRA, ZDHHC4) were further underscored by drug target MR and summary-data-based MR (SMR). Transcriptomics implicated dysregulation in the neuroactive ligand-receptor interactions and the G2/M DNA checkpoint pathway. Immune infiltration analysis demonstrated elevated CD4⁺ T cells and M0 macrophages in the high-LASSO score group. Integrated machine learning models built using the five key genes achieved robust predictive performance. Single-cell sequencing analysis confirmed gene expression patterns.

**Conclusion:** By integrating mediation MR, transcriptomics, and machine learning, this study demonstrated causal relationships between specific gut microbiota and CRC, with IBD as a mediator. We identified potential therapeutic targets and developed robust predictive models, providing crucial insights into the pathogenesis and clinical detection of CRC.

## Introduction

CRC is the third most commonly diagnosed malignancy and the second leading cause of cancer-related mortality worldwide. In the United States alone, more than 154,270 new CRC cases are projected to be diagnosed in 2025 1, 2. Despite advances in surgical methods, chemotherapy, and immunotherapy, the median survival for patients with advanced CRC remains poor, underscoring the urgent need for effective early detection and treatment.

IBD is a chronic inflammatory disorder of the gastrointestinal tract, which includes Crohn's disease and ulcerative colitis. Over recent decades, its prevalence has been steadily rising in Western countries. The incidence of early-onset IBD (diagnosed before the age of 20) is also increasing worldwide 3. IBD is a complex, multifactorial condition influenced by genetic susceptibility, immune dysregulation, environmental exposures, and alterations in the gut microbiome. Numerous epidemiological studies have demonstrated a significant association between IBD and an elevated risk of CRC, with chronic intestinal inflammation playing a central role in carcinogenesis 4.

In recent years, the gut microbiota has received increasing attention for its crucial role in the development of both IBD and CRC. As a key component of the intestinal ecosystem, it functions as a primary defense against pathogenic invasion and plays a pivotal role in maintaining mucosal homeostasis 5, 6. However, microbial dysbiosis may lead to inflammation, the production of bacterial toxins, and a compromise of the intestinal barrier integrity, thereby increasing the risk of IBD and CRC 7. Observational studies have reported that specific pathogenic bacteria, such as *enterotoxigenic Bacteroides fragilis* and pks^+^* Escherichia coli*, are more frequently found in CRC patients 8. In a pivotal study by Salahshouri *et al.*, the CRC-associated microbiome can promote inflammation and cancer progression through alterations in certain metabolites such as histamine, glutamine, and pyruvate, underscoring the functional contribution of microbiota in inflammatory responses and tumorigenesis 9.

Although existing evidence highlights the involvement of the gut microbiota in both IBD and CRC, most studies are observational and rely on limited datasets. The precise causal and mediating relationships among gut microbiota, IBD, and CRC remain poorly understood. Elucidating these connections is essential for developing targeted therapies and personalized prevention strategies.

MR is a powerful statistical method that leverages genetic variants as instrumental variables (IVs) to infer causal relationships between exposures and outcomes. The integration of multi-omics data, encompassing transcriptomics and immune infiltration analysis, with machine learning techniques has demonstrated significant potential in clarifying pathogenesis and enhancing risk prediction. In previous studies, Long Wu *et al.* employed MR to identify five gut microbial genera causally associated with CRC 10, while Martyna Pawlak *et al.* investigated differentially expressed genes (DEGs) and immune cell infiltration patterns in both IBD and CRC 11. However, no studies to date have systematically examined whether IBD mediates the causal pathway from gut microbiota to CRC. Furthermore, there remains a lack of integrated, multi-omics studies that leverage machine learning algorithms to unravel underlying mechanisms and construct predictive models for CRC.

**Figure 1:**
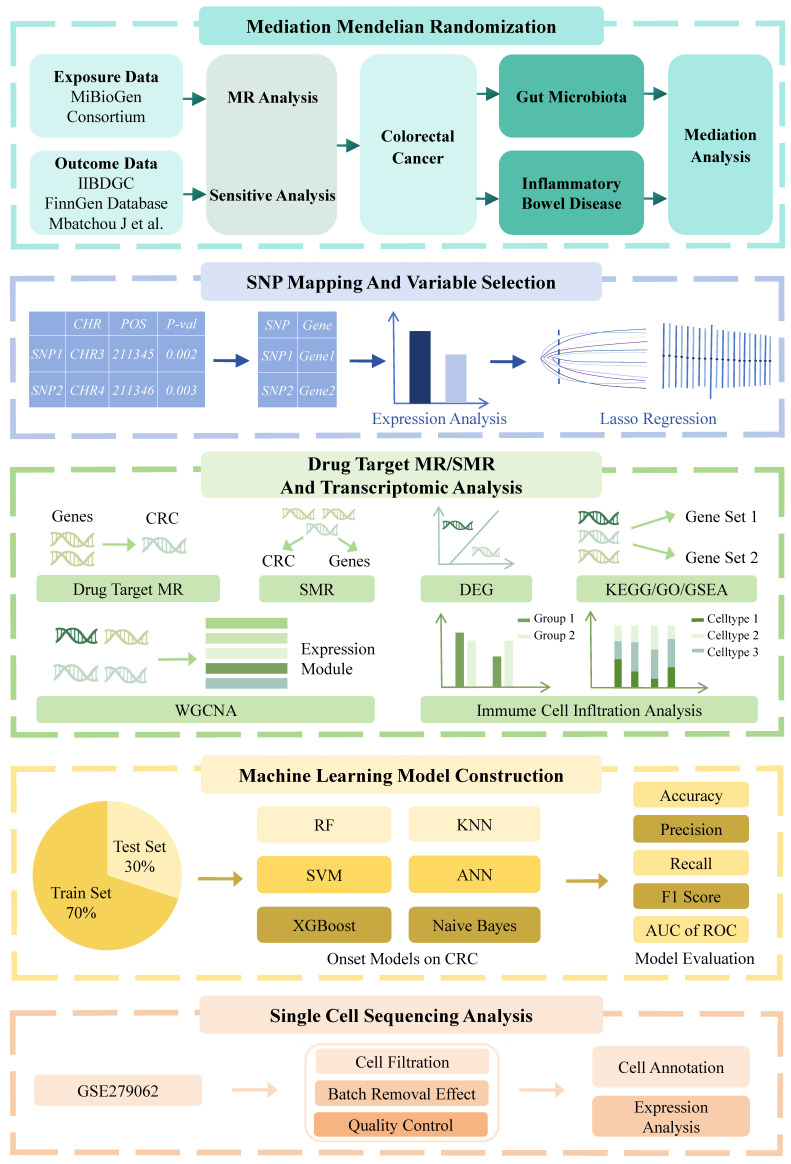
A flowchart of the study. This study was divided into five main components. The first component seeks to clarify the causal links between the gut microbiota, IBD, and CRC using mediation MR analysis. Then, SNP mapping to genes, variable selection, and drug target MR approaches were utilized to identify genes significantly correlated with CRC progression. Transcriptomic analysis was conducted to elucidate CRC-related pathways and molecular mechanisms. Subsequently, machine learning models were constructed using six machine learning algorithms on a training set (70%) and validated on a test set (30%). Finally, single-cell sequencing analysis is performed on dataset GSE279062, involving cell filtration, batch removal effect, quality control, and expression analysis to annotate cells and validate therapeutic targets. Abbreviations: IIBDGC, International Inflammatory Bowel Disease Genetics Consortium; MR, Mendelian randomization; SNP, Single nucleotide polymorphism; CHR, chromosome; POS, single nucleotide polymorphism position; Lasso, least absolute shrinkage and selection operator; SMR, summary-based Mendelian randomization; CRC, colorectal cancer; DEG, differentially expressed gene; GO, gene ontology; KEGG, Kyoto Encyclopedia of Genes and Genomes; GSEA, gene set enrichment analysis; WGCNA, weighted gene co-expression network analysis; RF, random forest; kNN, k-nearest neighbor; SVM, supporting vector machine; ANN, artificial neural network; XGBoost, extreme gradient boosting; AUC, area under the curve; ROC, receiver operating characteristic curve.

To address these knowledge gaps, we conducted a comprehensive analysis to elucidate the causal and mediating relationships among gut microbiota, IBD, and CRC using mediation MR methods. We mapped SNPs to genes and employed drug target MR to identify genes significantly associated with CRC progression. Transcriptomic analyses were conducted to investigate the biological functions and pathways. Six machine learning algorithms were applied to develop and validate predictive models for CRC. Additionally, single-cell sequencing analysis was employed to characterize cell-type-specific expression patterns of key genes (Figure 1). This integrative approach provides new insights into the microbiota-IBD-CRC axis and identifies potential therapeutic targets and prediction models for clinical translation.

## Materials and Methods

### Data source

Genetic data related to the gut microbiota were obtained from the MiBioGen consortium, which integrates 16S rRNA sequencing and SNP array data from approximately 19,000 individuals across 18 cohorts 12. The dataset includes 211 microbial taxa, encompassing 131 genera, 35 families, 20 orders, 16 classes, and 9 phyla. GWAS summary statistics for CRC were sourced from two large-scale studies: one by Huyghe JR *et al.*, involving 4,439 CRC cases and 4,115 controls of European ancestry, and another by Ishigaki K *et al.*, comprising 6,692 cases and 27,178 controls of East Asian ancestry 13, 14. GWAS data for IBD were drawn from three datasets of European ancestry: the International Inflammatory Bowel Disease Genetics Consortium (IIBDGC; 12,882 cases and 21,770 controls), the FinnGen study (5,673 cases and 213,119 controls), and the dataset published by Mbatchou J *et al.* (4,101 cases and 480,497 controls) 15, 16. Expression quantitative trait loci (eQTL) data were acquired from the initial phase of the eQTLGen consortium, which analyzed whole blood transcriptomes of 31,684 individuals to identify both cis- and trans-eQTLs, revealing associations between genetic variation and gene expression 17. Bulk RNA sequencing data for CRC were retrieved from The Cancer Genome Atlas (TCGA) CRC cohort. In addition, single-cell sequencing data were obtained from the study by Kabiljo J *et al.* (GSE279062), accessible through the NCBI Gene Expression Omnibus, comprising single-cell transcriptomic profiles from seven CRC patients.

### Selection of IVs

**Figure 2:**
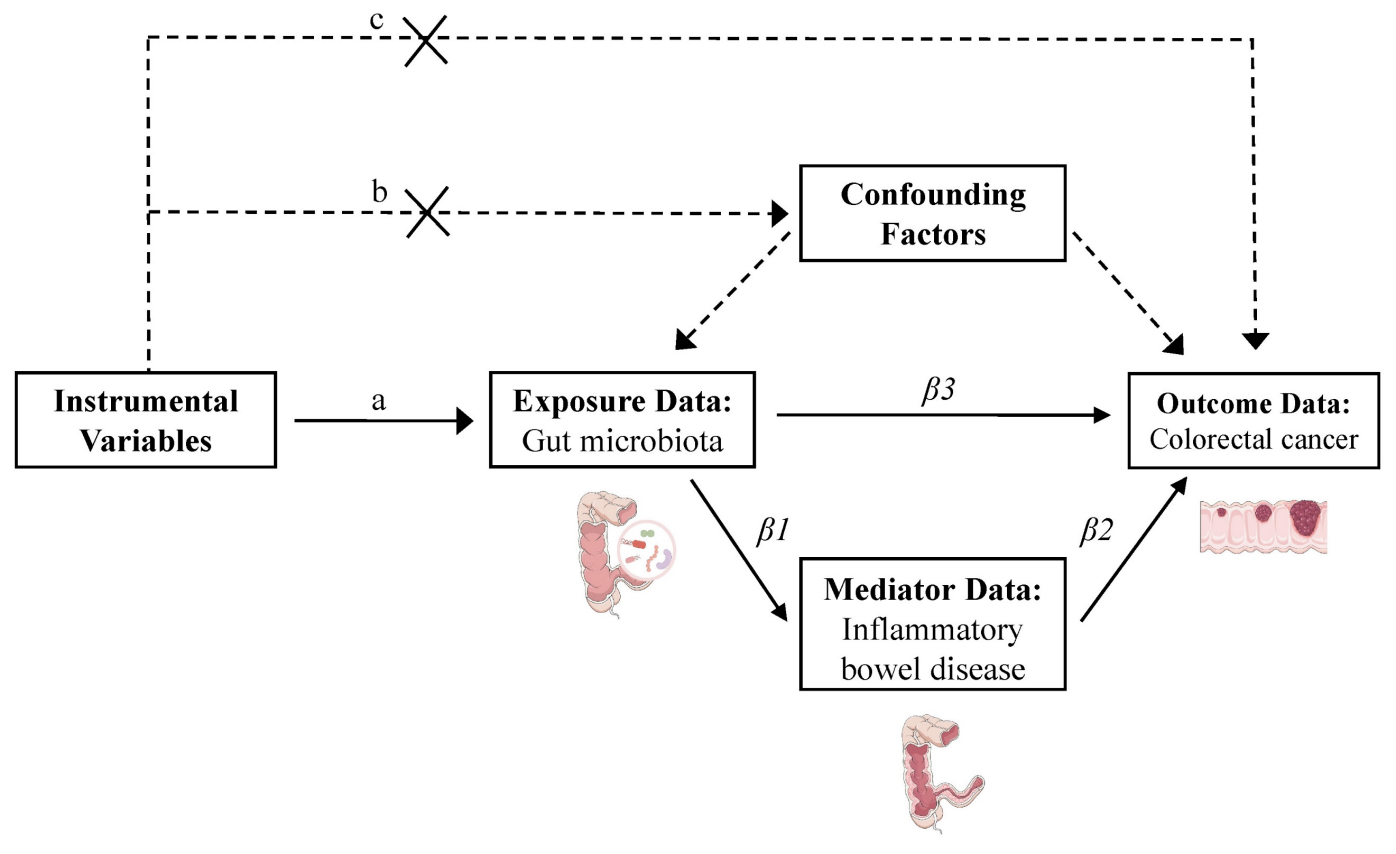
Flowchart of the mediation Mendelian randomization and its three key assumptions. (a) Relevance assumption: The instrumental variables must be associated with the exposure. (b) Independence assumption: The instrumental variables should not influence outcomes through factors other than exposure. (c) Exclusivity assumption: The instrumental variables should not influence outcomes directly. Effect estimates are defined as follows: β1 represents the causal effect of gut microbiota on inflammatory bowel disease; β2 denotes the causal effect of inflammatory bowel disease on colorectal cancer; and β3 corresponds to the total effect of gut microbiota on colorectal cancer. The proportion mediated by inflammatory bowel disease is calculated as: mediation effect proportion = (β1 × β2) / β3.

To ensure the validity of MR analysis, SNPs selected as IVs were required to satisfy three core assumptions 18: 1) Relevance assumption: the SNPs must be strongly associated with the exposure. 2) Independence assumption: the SNPs must be independent of confounders and the outcome; and 3) Exclusivity assumption: the SNPs only influence the outcome through the exposure. To meet these assumptions, we applied the following stringent selection criteria: 1) SNPs with a p-value < 5 × 10⁻⁶ for SNP-exposure associations were retained to maximize the number of eligible variants; 2) SNPs were pruned for linkage disequilibrium using a threshold of r² < 0.01 and a window size > 10,000 kb 19; 3) SNPs with p-values > 0.05 for association with the outcome were excluded to minimize confounding; 4) SNPs with a minor allele frequency < 0.01 were removed 20; 5) Proxy SNPs were excluded. 6) All SNPs were harmonized for effect allele orientation, and palindromic SNPs were removed; 7) SNPs with F-statistics ≤ 10 were excluded to mitigate weak instrument bias 6 (Figure 2).

The proportion of exposure variance explained by IVs (R^2^) was calculated using the formula: R^2^ = 2 × EAF × (1 - EAF) × β^2^ / (2 × EAF × (1 - EAF) × β^2^ + 2 × EAF × (1 - EAF) × N × SE^2^), where EAF is the effect allele frequency, β is the effect size between the SNP and the exposure trait, SE is the variance of β, and N is the sample size. Instrument strength was further assessed using the F-statistic, calculated as F = R^2^ × (N - 2) / (1 - R^2^) 21.

### MR analysis

#### Preliminary analysis

We conducted two-sample MR analyses to estimate the causal association between IBD and CRC, as well as between gut microbiota and CRC. For bacterial taxa associated with a single SNP, causal inference was performed using the Wald ratio method, calculated as the ratio of the SNP-outcome effect to the SNP-exposure effect. When multiple SNPs were available, five complementary MR methods were applied: inverse variance weighting (IVW), MR-Egger regression, weighted median, weighted mode, and simple mode. Among these, IVW was used as the gold standard to provide a more conservative but robust estimate 22.

Although the IVW method is frequently employed due to its statistical efficiency, it can produce biased outcomes in the presence of horizontal pleiotropy 23. In order to ensure the robustness of our findings, we included MR-Egger and weighted median methods as complementary methods. The MR-Egger regression yields unbiased estimates even in the presence of directional pleiotropy, although it is susceptible to outliers 24. The weighted median method provides consistent results if at least 50% of the IVs are valid 25. The weighted mode method assumes that the largest subset of instruments with similar causal estimates is valid, making it particularly useful in settings with heterogeneity among IVs. The simple mode method, which does not weight instruments, estimates causal effects by identifying clusters of IVs with consistent directional effects. Although this method demonstrates relative robustness to heterogeneity, it is generally less efficient and may perform suboptimal results, particularly when the number of IVs is limited 26.

Results from the MR analyses were reported as regression coefficients, standard errors, odds ratios (ORs), and 95% confidence intervals. To control for false discovery rates due to multiple testing, p-values were modified using the Benjamini-Hochberg correction. Statistical significance was defined as a p-value < 0.05 and an adjusted p-value (p-adjusted) < 0.10 27.

#### Mediation MR analysis

In preliminary analyses, we performed two-sample MR to identify gut microbial taxa and IBD datasets associated with CRC. To assess whether IBD mediates the relationship between gut microbiota and CRC, we subsequently conducted a two-step MR mediation analysis. First, IBD datasets that showed positive associations with at least one CRC dataset were retained to maximize the inclusion of relevant SNPs. The causal effects of IBD on CRC (β2 in Figure 2) were then estimated using two-sample MR 28. Second, we identified microbial taxa that exhibited consistent causal associations with all selected IBD datasets and calculated their effects on IBD (β1 in Figure 2) via two-sample MR. The total effects of these taxa on CRC (β3 in Figure 2) were obtained from the initial microbiota-CRC MR analysis. The mediation effect was estimated using the product of coefficients method as (β1 × β2). The direct effect was calculated as (β3 - β1 × β2), and the proportion of the total effect mediated by IBD was computed as (β1 × β2) / β3 29.

#### Sensitivity analysis

We performed several sensitivity analyses to address potential pleiotropy. Cochran's Q test was used to assess heterogeneity across SNP-specific effect estimates 30. Significant Q values (p-value < 0.05) indicate the presence of heterogeneity, suggesting potential variability in causal effects. A leave-one-out analysis was conducted by sequentially excluding each SNP and recalculating the MR estimate based on the remaining instruments, thereby evaluating the impact of each SNP on the results. MR-Egger intercept analysis was employed to detect horizontal pleiotropy using the intercept term. An intercept close to 0 suggests no horizontal pleiotropy, supporting the validity of the IVs 31. MR-Steiger test was applied to confirm the causal direction, determining whether the observed relationships are more consistent with the hypothesis that the exposure influences the outcome rather than the reverse 32.

#### SNP mapping to genes

We identified SNPs from gut microbiota that exhibited consistent causal associations across all IBD and CRC datasets. These SNPs were subsequently mapped to their corresponding genes using PLINK, a widely used open-source software suite for analyzing genotypic and phenotypic data. PLINK enables SNP annotation based on physical proximity to gene regions, thereby facilitating accurate genomic localization and identifying SNP-gene relationships 33. Only SNPs located within gene regions were retained for further analysis, and a gene list was compiled accordingly to elucidate the genetic influences of gut microbiota on IBD and CRC.

#### Expression analysis and variable selection

Gene expression data from the TCGA database were utilized to compare the expression levels of previously identified genes between CRC and adjacent normal tissue samples. Differential expression analysis was conducted using the Wilcoxon rank-sum test 34. Statistical significance was defined as p-value < 0.05 and p-adjusted < 0.10 after multiple testing correction 35.

To identify the genes most strongly associated with CRC, we constructed a predictive model using the LASSO regression. LASSO is a linear regression technique that applies L1 regularization to perform variable selection and control model complexity. By introducing a penalty term (λ), the method shrinks regression coefficients; as λ increases, more coefficients are reduced, with some ultimately compressed to zero, which helps in selecting important features and reducing overfitting. The optimal λ value was determined using 10-fold cross-validation by evaluating both the minimum mean squared error (λ-min) and one standard error above the minimum (λ-min + 1se). λ-min usually selects more variables and results in a more complex model, but provides the best prediction performance. In contrast, λ-min + 1se yields a sparser model with potentially reduced performance. In this study, we selected λ-min to maximize model accuracy while preserving more informative genes 36. The resulting regression coefficients reflect the relative contribution of each gene to disease classification. A LASSO score model was then constructed by calculating the weighted sum of gene expression levels and their corresponding coefficients, quantifying individual CRC risk 37.

#### Drug target MR and SMR analysis

Drug target MR utilizes genetic variants associated with drug target genes as IVs to infer causal relationships between gene expression regulation and disease outcomes. In this study, we used eQTL data from the first phase of the eQTLGen Consortium for genes identified through LASSO regression as the exposure dataset, and CRC GWAS data as the outcome dataset. The IVW method served as the gold standard for testing causal relationships between gene expression and CRC risk 38.

To validate and complement these results, we used the SMR software, which was developed to implement the SMR and Heterogeneity in Dependent Instruments (HEIDI) methods. It integrates eQTL and GWAS summary statistics to identify pleiotropic associations between gene expression and disease traits. For each gene, the most significantly associated cis-eQTL SNP was selected as the top IV. The primary outcome was presented as the OR for CRC per standard deviation increase in gene expression. To distinguish true causal associations from those confounded by linkage or pleiotropy, we conducted the HEIDI test 39. A HEIDI p-value < 0.05 suggests that the observed association may be due to multiple causal variants or complex gene regulation rather than a single causal pathway. Genes with SMR p-values < 0.10 and HEIDI p-values > 0.05 are considered to have robust and potentially causal associations with CRC risk 40.

### Transcriptome analysis

To systematically explore transcriptional patterns associated with CRC, we applied performed weighted gene co-expression network analysis (WGCNA), a systems biology approach that identifies modules of highly co-expressed genes based on their correlation patterns and associations with clinical traits 41. All analyses were performed using the WGCNA R package. First, Pearson correlation coefficients were computed for all gene pairs to quantify expression similarity. A suitable soft-thresholding power was then selected to transform the correlation matrix into an adjacency matrix, reflecting the strength of the connections between genes. Next, a topological overlap matrix was constructed to quantify the degree of gene interconnectivity. Hierarchical clustering combined with dynamic tree cutting was applied to detect gene modules with similar expression patterns or potential functional relevance 32. Among the identified modules, the three most significantly correlated with CRC onset were retained for downstream analyses.

DEG analyses were conducted using data from the TCGA CRC dataset to compare gene expression profiles between CRC patients and normal controls. A second DEG analysis was performed by stratifying samples based on LASSO-derived scores from previously identified CRC-associated genes. Samples were divided into high- and low-LASSO score groups using the median LASSO score as the cutoff. For both analyses, p-values were adjusted for multiple testing, and genes with p-adjusted < 0.10 were retained. These genes were ranked in descending order by the absolute value of their log_2_ fold change, and the top 2,500 ranked genes from each analysis were intersected with WGCNA-derived genes for enrichment analysis. To interpret the biological functions of these intersecting genes, Gene Ontology (GO) 42, Kyoto Encyclopedia of Genes and Genomes (KEGG) pathway analysis 43, and Gene Set Enrichment Analysis (GSEA) 44 were conducted.

Additionally, immune infiltration analysis was carried out on the high- and low-LASSO score groups utilizing the CIBERSORT in R. This allowed us to investigate whether the differential gene expression changes were associated with changes in specific immune cell populations, providing further insight into potential immunological mechanisms underlying CRC pathogenesis.

### Construction of a CRC onset model based on machine learning

The TCGA CRC dataset was randomly split into training and test sets at a ratio of 7:3. Predictive models were developed using the training set with six machine learning algorithms: random forest (RF), support vector machines (SVM), extreme gradient boosting (XGBoost), artificial neural networks (ANN), naive Bayes, and k-nearest neighbors (kNN). Hyperparameters were tuned with the caret package in R to maximize the area under the receiver operating characteristic curve (AUC).

RF aggregates multiple decision trees to improve accuracy in classification or regression and minimize overfitting 45. SVM constructs an optimal hyperplane in a high-dimensional space to separate classes while maximizing the margin between data points and decision boundaries 46. ANN consists of multiple layers of interconnected artificial neurons that process input data via weighted summation and activation functions, enabling the modeling of complex, nonlinear patterns 47. XGBoost is a powerful gradient boosting algorithm that iteratively builds an ensemble of weak learners, typically decision trees, while minimizing the loss function through gradient descent 48. kNN is a non-parametric, instance-based learning method that calculates Euclidean distances to training set points, selects the k nearest neighbors, and predicts outputs based on their labels or values 49. Naive Bayes is a probabilistic classifier grounded in Bayes' theorem, which assumes conditional independence between features 50.

Model performance was evaluated on the test set using five metrics: AUC, sensitivity, specificity, recall, and F1 score 51. To assess the robustness and generalizability of each model, we performed 400 iterations of 10-fold cross-validation and visualized the resulting AUC distributions using boxplots. Although the machine learning algorithms used in this study are widely applied in existing clinical risk prediction, the optimal model may vary depending on disease characteristics and population heterogeneity. To enhance predictive stability, we employed a soft voting ensemble strategy that integrated the probability outputs from all models. Each model generated a probability score ranging from 0 to 1, with higher values indicating greater predicted CRC risk. The final prediction was obtained by averaging these probabilities. This ensemble approach leveraged the complementary strengths of individual algorithms, thereby enhancing overall predictive accuracy and stability 52.

### Single-cell sequencing analysis

Single-cell sequencing provides detailed insights into gene expression at the individual cell level by combining cell isolation, library preparation, high-throughput sequencing, and advanced computational analysis techniques 53. In this study, we analyzed single-cell sequencing data from CRC patients as reported by Kabiljo J *et al.*, to construct cell-type-specific gene expression profiles within the immune system and tumor microenvironment 54. The GSE279062 dataset from the comprehensive gene expression database (GEO, https://www.ncbi.nlm.nih.gov/geo/) was downloaded for analysis. This cohort includes cancer-associated fibroblasts (CAFs) and primary monocytes or macrophages derived from 7 CRC patients. The sequencing matrix data were integrated using the R software package “Seurat”. Cell filtering criteria were set to exclude cells expressing fewer than 300 or more than 5,000 genes, with mitochondrial gene content kept below 10% and red blood cell gene content below 1%. Additionally, only cells with a unique molecular identifier count greater than 600 were retained. The Seurat package in R was utilized for cell annotation and identification of distinct cellular populations, elucidating CRC pathogenesis and potential therapeutic targets 55. Subsequently, the data were normalized and standardized. Highly variable genes are selected for dimensionality reduction through principal component analysis, followed by batch correction using the Harmony algorithm. Clustering was performed, and the results were visualized using Uniform Manifold Approximation and Projection, with cell clusters annotated according to known marker genes.

## Results

### Selection of IVs

Based on predefined selection criteria, we identified 658, 1117, 1039, 1624, and 4342 SNPs associated with 211 gut microbiota taxa at the phylum, class, order, family, and genus levels, respectively. In total, 8,780 SNPs were selected as IVs for downstream MR analyses of these microbial classifications (Table S12). For IBD, 1,596, 1,626, and 1,636 SNPs were selected as IVs across three GWAS datasets (Tables S13-15). For CRC, 1,637 and 1,343 IVs were identified from two datasets (Tables S16-17).

### MR analysis

#### The causal effect of IBD on CRC

A two-sample MR analysis was conducted to evaluate the causal relationship between IBD and CRC. IVs derived from the FinnGen and IIBDGC datasets were significantly associated with CRC risk in the Huyghe JR *et al.* dataset (FinnGen: P-value = 0.028; IIBDGC: P-value = 0.017). Similarly, IVs from Mbatchou J *et al.* were significantly associated with CRC risk in the Ishigaki *et al.* datasets (P-value = 0.006). To maximize analytical power and biological interpretability, we retained all significant associations for downstream analysis following sensitivity analyses. Detailed results can be found in Table S4.

#### Causal effects of gut microbiota on CRC and IBD

A two-sample MR analysis was performed to investigate the causal effects of gut microbiota on both CRC and IBD. After multiple testing correction, 101 and 90 taxa exhibited causal relationships with CRC in the GWAS datasets by Huyghe JR *et al.* and Ishigaki *et al.*, respectively. Likewise, 82, 99, and 83 microbial taxa were identified as causally associated with IBD in the FinnGen, IIBDGC, and Mbatchou J *et al.* datasets, respectively. By intersecting the taxa significantly associated with both CRC and IBD, 13 overlapping taxa were identified, including 1 phylum, 1 class, 1 order, 2 families, and 8 genera. However, the genus *LachnospiraceaeND3007 group* was excluded from further analysis due to insufficient statistical power (power < 0.5), which could compromise the reliability of causal inference. The remaining 12 taxa were retained for downstream analyses, suggesting potential shared causal roles in both CRC and IBD. To assess the robustness of these findings, sensitivity analyses were performed, with detailed results provided in Tables S1-S3 and S5-S6. Based on the consistency across multiple MR methods, current literature support, and the biological plausibility of the identified taxa, all 12 were included in subsequent analyses.

In our analysis, the genetically predicted relative abundance of nine microbial taxa showed consistent protective or pathogenic effects on CRC across both GWAS datasets. A higher abundance of the phylum *Actinobacteria* was associated with reduced CRC risk (Huyghe JR *et al.*: OR = 0.844, 95% CI: 0.791-0.901; Ishigaki K *et al.*: OR = 0.913, 95% CI: 0.879-0.948). Similar protective associations were observed for its subordinate taxa, including class* Actinobacteria* (Huyghe JR *et al.*: OR = 0.843, 95% CI: 0.810-0.876; Ishigaki K *et al.*: OR = 0.903, 95% CI: 0.876-0.930), family* Bifidobacteriaceae* (Huyghe JR *et al.*: OR = 0.889, 95% CI: 0.854-0.925; Ishigaki K *et al.*: OR = 0.914, 95% CI: 0.891-0.937), genus* Bifidobacterium* (Huyghe JR *et al.*: OR = 0.864, 95% CI: 0.831-0.898; Ishigaki K *et al.*: OR = 0.888, 95% CI: 0.865-0.912), and order* Bifidobacteriales* (Huyghe JR *et al.*: OR = 0.889, 95% CI: 0.854-0.925; Ishigaki K *et al.*: OR = 0.914, 95% CI: 0.891-0.937). Other taxa demonstrating protective associations included genera *Streptococcus* (Huyghe JR *et al.*: OR = 0.415, 95% CI: 0.384-0.449; Ishigaki K *et al.*: OR = 0.845, 95% CI: 0.745-0.959) and *Lachnoclostridium* (Huyghe JR *et al.*: OR = 0.604, 95% CI: 0.518-0.704; Ishigaki K *et al.*: OR = 0.809, 95% CI: 0.725-0.901). In contrast, elevated genetically predicted abundance of the genera *Eubacterium coprostanoligenes group* (Huyghe JR *et al.*: OR = 1.610, 95% CI: 1.358-1.910; Ishigaki K *et al.*: OR = 1.222, 95% CI: 1.118-1.336) and *RuminococcaceaeUCG011* (Huyghe JR *et al.*: OR = 1.371, 95% CI: 1.314-1.430; Ishigaki K *et al.*: OR = 1.249, 95% CI: 1.203-1.297) was associated with an increased risk of CRC.

Regarding IBD, seven microbial taxa demonstrated consistent protective or pathogenic effects across three IBD datasets. These included the phylum* Actinobacteria*, its subordinate class* Actinobacteria*, *Bifidobacteriaceae*, *Bifidobacterium*, *Bifidobacteriales*, as well as *Eubacterium coprostanoligenes group* and *RuminococcaceaeUCG011*. Among these, phylum* Actinobacteria* was found to be the most protective, consistently demonstrating significant effects across all datasets (FinnGen: OR = 0.765, 95% CI: 0.734-0.797; IIBDGC: OR = 0.721, 95% CI: 0.695-0.748; Mbatchou J *et al.*: OR = 0.995, 95% CI: 0.995-0.996). In contrast, the *Eubacterium coprostanoligenes group* showed the strongest positive association with IBD risk across all datasets (FinnGen: OR = 1.161, 95% CI: 1.027-1.313; IIBDGC: OR = 1.320, 95% CI: 1.201-1.451; Mbatchou J *et al.*: OR = 1.006, 95% CI: 1.005-1.007). Importantly, the taxa that conferred risk or protection for IBD demonstrated similar roles in CRC, supporting the hypothesis of shared microbiota-mediated mechanisms underlying both gastrointestinal diseases (Tables 1 and 2).

#### Mediating effects of IBD on gut microbiota-CRC relationship

We applied a two-step MR method to investigate the mediating role of IBD in the causal relationship between 12 gut microbial taxa and CRC. The proportion of mediation varied across different microbial classifications, as summarized in Table 3.

In the CRC dataset from Huyghe JR *et al.*, IBD (FinnGen dataset, β = -0.107, P-value = 0.028) mediated the relationship between *Alcaligenaceae* (β = 0.693, P-value = 9.732E-26) and CRC (Huyghe JR *et al.*) with a mediation effect of -0.026, accounting for 3.8% of the total effect. Likewise, using the IIBDGC dataset (β = -0.062, P-value = 0.017), IBD was found to mediate the relationships between *Streptococcus*, an unknown genus (id.1868), and *Peptococcus* with CRC, with corresponding mediation effects of -0.021 (2.44%), -0.004 (3.082%), and -0.009 (12.007%), respectively.

In the Ishigaki K *et al.* CRC dataset, IBD (Mbatchou J *et al.*) mediated the relationship between class* Actinobacteria* (β = -0.102, P-value = 9.839E-12), *Bifidobacteriaceae* (β = -0.090, P-value = 1.792E-12), *Eubacterium coprostanoligenes group* (β = 0.200, P-value = 1.063E-05), *Bifidobacterium* (β = -0.119, P-value = 1.470E-18), *RuminococcaceaeUCG011* (β = 0.222, P-value = 2.746E-31), an unknown genus (id.1868, β = 0.079, P-value = 0.003), *Bifidobacteriales* (β = -0.090, P-value = 1.792E-12), and phylum* Actinobacteria* (β = -0.091, P-value = 2.564E-06) and CRC, with mediation effects of -0.038 (36.997%), -0.041 (45.678%), 0.064 (31.848%), -0.037 (30.857%), 0.014 (6.129%), 0.008 (10.232%), -0.041 (45.678%), and -0.051 (55.725%), respectively.

No mediation effects were observed for other taxa due to the inconsistent direction of their β values. Collectively, these findings provide evidence that IBD acts as a partial mediator in the causal pathway between specific microbial taxa and CRC development. Detailed results can be found in Table S7.

#### SNP mapping to genes

A total of 1,443 SNPs associated with the 12 gut microbial taxa were identified via MR analysis and mapped to corresponding genes using PLINK. SNPs located within gene regions were retained and annotated with gene symbols, shown as “GeneSymbolName(0)”, while those without gene annotations were excluded from subsequent analysis.

In the causal pathway where IBD (FinnGen dataset) mediated the association between *Alcaligenaceae* and CRC (Huyghe JR *et al.*), 5 genes were identified. In the causal pathways in which IBD (IIBDGC dataset) mediated the effects of *Streptococcus*, an unknown genus (id.1868), and *Peptococcus* on CRC (Huyghe JR *et al.*), 21 genes were identified. Likewise, in the causal pathways where IBD (Mbatchou J *et al.*) mediated the associations between class* Actinobacteria*, *Bifidobacteriaceae*, *Eubacterium coprostanoligenes group*, *Bifidobacterium*, *RuminococcaceaeUCG011*, an unknown genus (id.1868), *Bifidobacteriales*, and phylum* Actinobacteria* on CRC (Ishigaki K *et al.*), a total of 36 genes were identified. After merging gene lists and removing duplicates, a final set of 59 unique genes was obtained for downstream analyses. Detailed results are presented in Tables S18-S20.

#### Expression analysis and variable selection

**Figure 3:**
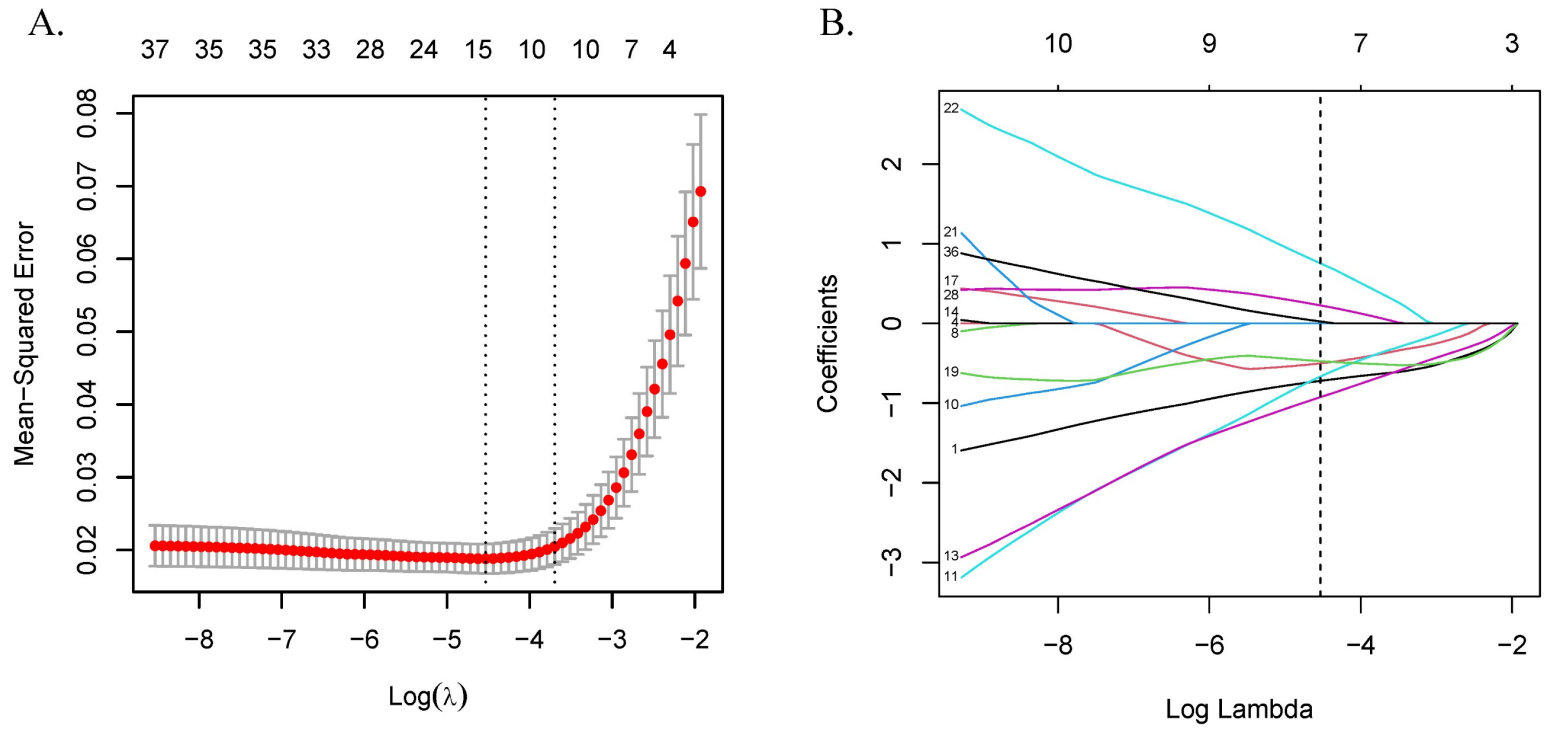
Variable selection using the LASSO algorithm. (A) LASSO cross-validation error plot. The plot illustrates the relationship between the mean square error and the logarithm of the regularization parameter (λ). The vertical dashed lines indicate the optimal values of λ determined by cross-validation: λ-min+1se and λ-min. (B) LASSO coefficient path plot. This plot shows the coefficient paths of each variable change as λ varies. The vertical solid line corresponds to the logarithm of the optimal λ (λ-min) we chose to identify the eight genes that best fit the LASSO model. Abbreviations: LASSO, least absolute shrinkage and selection operator.

Differential expression analysis between CRC and control groups identified 37 DEGs among the 59 candidates after adjusting the P-values. These genes were subsequently used to construct a LASSO regression model, with 10-fold cross-validation determining the optimal λ (λ-min = 0.011, ln(λ-min) = -4.533) and selecting eight genes for further analysis. The LASSO score for each sample was calculated using the formula score = Σ (gene coefficient × gene expression). Detailed results are presented in Table S8 and Figure 3.

#### Drug target MR and SMR analysis

Due to the unavailability of eQTL data for ADCYAP1R1, drug target MR analyses were conducted on the remaining seven candidate genes. In the dataset from Huyghe JR *et al.*, all seven genes showed significant associations with CRC risk. Specifically, elevated expression levels of ATP11A, GBE1, and SOAT1 were associated with an increased risk of CRC (OR > 1), while increased expression of FAM120A, MCM6, MSRA, and ZDHHC4 was associated with a reduced risk (OR < 1). In the Ishigaki K *et al.* dataset, four genes were significantly associated with CRC after P-value adjustment, among which FAM120A and MCM6 consistently demonstrated protective effects.

Given the potential confounding effects of linkage disequilibrium on causal inference between gene expression and CRC risk, we applied SMR and HEIDI tests to validate the drug target MR results. Due to data availability, SMR analysis was restricted to the Ishigaki K *et al.* dataset, which identified four genes meeting the predefined criteria for association with CRC (P-SMR < 0.10 and P-HEIDI > 0.05). Notably, increased expression of MSRA and ZDHHC4 was significantly associated with a decreased CRC risk, consistent with findings from the Huyghe JR *et al.* dataset. To enhance the robustness of downstream analyses, five genes—FAM120A, MCM6, MSRA, GBE1, and ZDHHC4—that demonstrated consistent associations in at least two of the drug target MR or SMR analyses were prioritized for further investigation. Detailed results can be found in Tables 4 and S9-11.

### Transcriptome analysis

**Figure 4:**
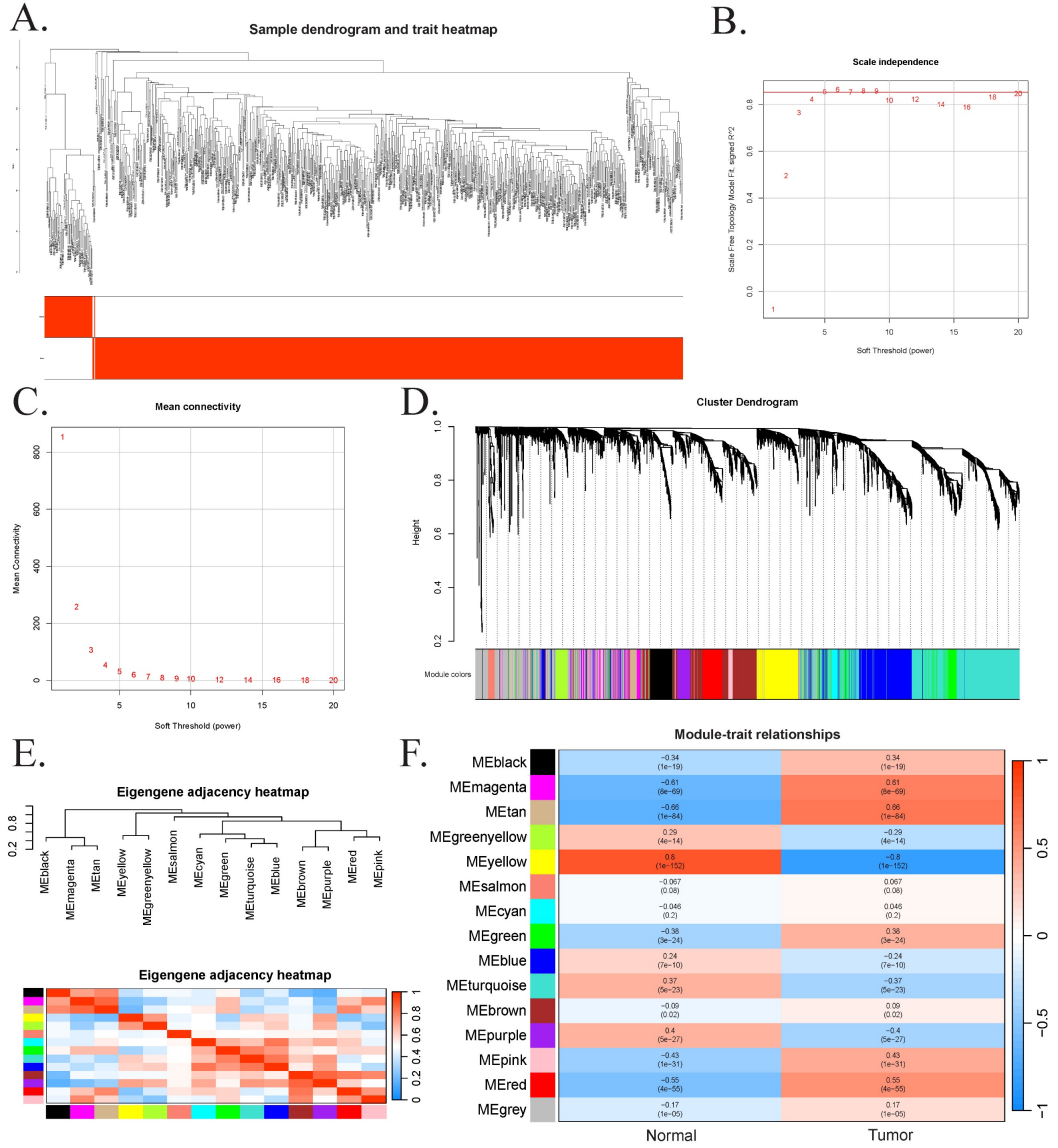
Results of weighted gene co-expression network analysis. (A) Sample clustering dendrogram, where each leaf represents a sample. (B) The impact of soft-threshold power on the scale-free topology fit index. A soft-thresholding power of β = 5 was selected, achieving a scale-free topology fit index (R2) of 0.85. (C) The impact of soft-threshold power on the mean connectivity. (D) Hierarchical cluster analysis of co-expression clusters with corresponding color assignments. (E). Collinear heat map of module feature genes. Red color represents a high correlation, and blue color represents the opposite trend. (F). The heatmap of the relationship between module eigengenes and clinical traits.

We applied WGCNA to investigate gene expression patterns in CRC patients by performing hierarchical clustering on all samples. After excluding one outlier sample (TCGA-AA-3695-01A) and filtering out low-expression genes, a total of 51 normal and 617 CRC samples were retained for analysis (Figure 4A). A soft-thresholding power of 5 was selected to ensure scale-free topology (R² = 0.85), and the corresponding mean connectivity plot was generated (Figure 4B-C). Co-expressed genes were grouped into modules using a dynamic tree-cutting algorithm, and modules with eigengene correlations greater than 0.75 were merged. This process identified 15 co-expression modules, each labeled by a distinct color and comprising at least 52 genes (Figure 4D-E). Among these, the magenta, tan, and red modules showed the strongest correlations with CRC pathogenesis, contributing 513 genes to downstream analyses (Figure 4F).

**Figure 5:**
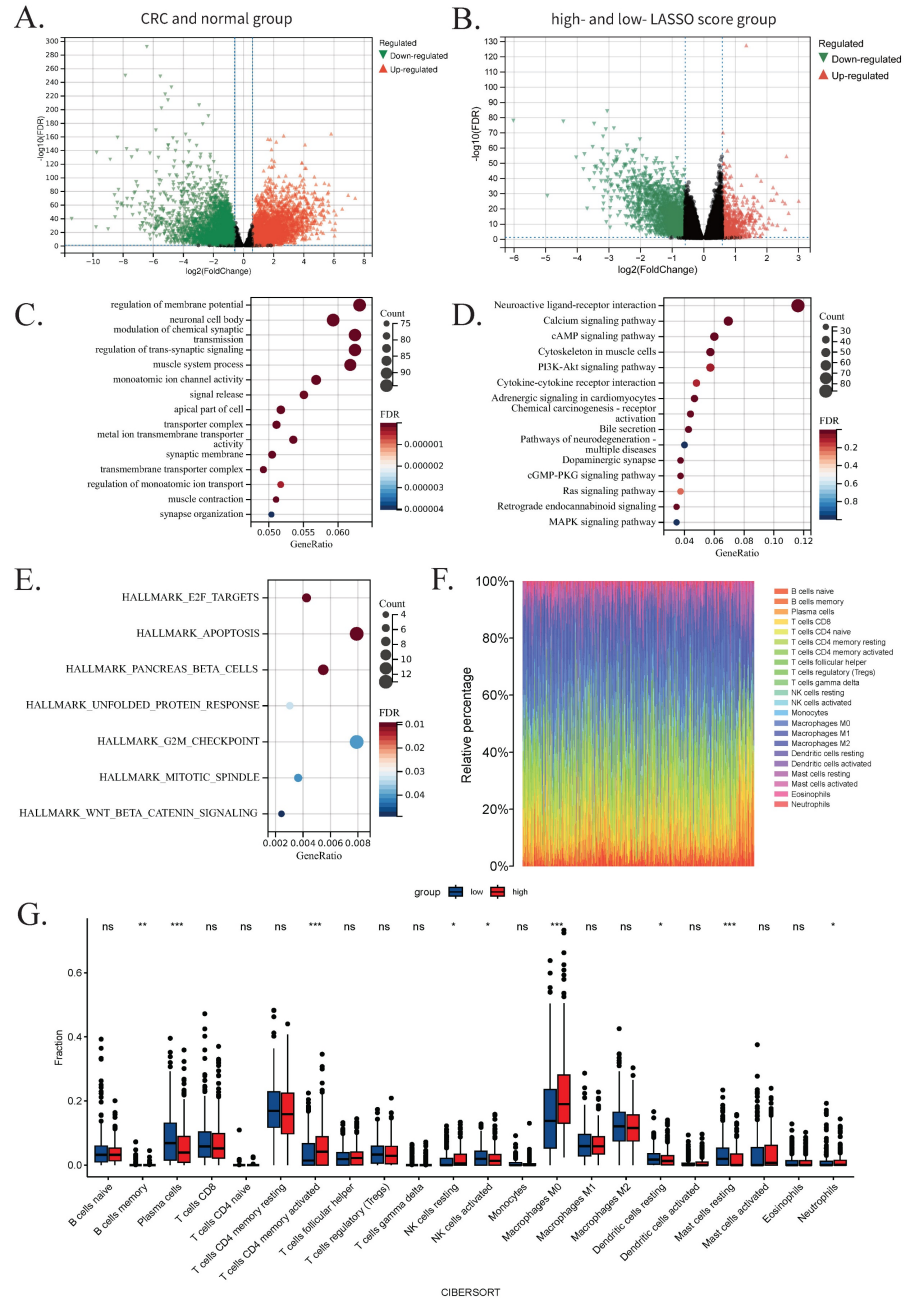
Results of transcriptome analysis. (A) Volcano plot showing 2,500 differentially expressed genes between the CRC group and the normal group. (B) Volcano plot showing 2,500 differentially expressed genes between the high- LASSO score group and the low-LASSO score group. (C) Bubble plots of Gene Ontology analysis results for the 1,666 intersected genes. (D) Bubble plots of Kyoto Encyclopedia of Genes and Genomes analysis results for the 1,666 intersected genes. (E) Bubble plots of Gene Set Enrichment Analysis results for the 1,666 intersected genes. (F) Rainbow plot showing the proportions of 22 immune cells in the low-LASSO and high-LASSO groups estimated by CIBERSORT. (G) Grouped bar chart illustrating a comparison of immune-cell infiltration scores between the low-LASSO and high-LASSO score groups estimated by CIBERSORT. Statistical significance: ns, not significant; *, P-value<0.05; **, P-value<0.01; ***, P-value<0.001. Abbreviations: CRC, colorectal cancer; LASSO, least absolute shrinkage and selection operator.

Differential expression analyses were performed both between CRC and normal samples, as well as between high- and low-LASSO score groups based on previously identified CRC-related genes (Figure 5A-B). After excluding missing values and adjusting for multiple testing, the top 2,500 genes were selected and ranked by absolute log_2_ Fold Change. Intersecting the DEG results with WGCNA module genes yielded 1,666 overlapping genes, which were subjected to functional enrichment analysis.

GO analysis revealed significant enrichment in biological processes such as regulation of membrane potential, neuronal cell body, and modulation of chemical synaptic transmission. KEGG analysis indicated enrichment in neuroactive ligand-receptor interaction, calcium signaling, and cAMP signaling pathways. GSEA analysis further highlighted enrichment in pathways including apoptosis, G2/M checkpoint, and pancreatic β-cell function. The top 15 GO and KEGG terms and all GSEA results are presented in Figure 5C-E.

To explore the role of immune cell infiltration in CRC progression, we used CIBERSORT to estimate immune cell proportions between high- and low-LASSO score groups. Rainbow plots visualized the composition of immune cell subsets across samples (Figure 5F). Subgroup bar plots demonstrated significant differences in the levels of 22 immune cell types, underscoring the heterogeneity of immune responses in CRC. Notably, nine cell types exhibited statistically significant differences: memory B cells, plasma cells, activated NK cells, resting dendritic cells, and resting mast cells were more abundant in the low-LASSO score group; whereas activated CD4⁺ memory T cells, resting NK cells, M0 macrophages, and neutrophils were more enriched in the high-LASSO score group (Figure 5G).

### Construction of a CRC onset model based on machine learning

Six machine learning models were developed to predict CRC status based on five key genes identified through drug-target MR and SMR analyses, with the aim of simplifying the model structure and enhancing prediction efficiency and accuracy.

The RF model was developed utilizing the randomForest package, with optimized hyperparameters: mtry (number of features sampled per split) = 3, and ntree (number of decision trees) = 500. In the test set, the model achieved an accuracy of 0.975, precision of 0.952, recall of 1.000, F1 score of 0.975, and an AUC of 0.900. Feature importance, assessed via the Mean Decrease Gini index, identified MCM6 as the most influential gene (Figure S1B). The SVM model, implemented using the e1071 package with an eps-regression kernel, was configured with cost = 10, gamma = 0.01, and epsilon = 0.1. This model also exhibited outstanding performance, with an accuracy of 0.975, a precision of 0.952, a recall of 1.000, an F1 score of 0.975, and AUC of 0.972.

The ANN model, constructed with two hidden layers comprising 2 and 6 neurons, respectively, attained an accuracy of 0.911, a precision of 0.930, a recall of 0.889, an F1 score of 0.909, and an AUC of 0.931. Feature importance was evaluated using the neuralnet package, which calculates the relative contribution of each gene based on internal weight parameters. Consistent with the RF model, MCM6 was identified as the most predictive feature, which demonstrates the robustness of our results (Figure S1E). The XGBoost model was implemented using the XGBoost package with optimum parameters: nrounds = 300, max_depth = 3, eta = 0.1, gamma = 0, and colsample_bytree = 0.8. It achieved an accuracy of 0.967, a precision of 0.937, a recall of 1.000, an F1 score of 0.967, and an AUC of 0.952.

The kNN model, also developed using the e1071 package, employed k = 19 as the optimal number of nearest neighbors. This model achieved an accuracy of 0.920, a precision of 0.947, a recall of 0.889, an F1 score of 0.917, and an AUC of 0.943. The Naive Bayes model, also constructed using the e1071 package with a Laplace smoothing parameter of 0, attained an accuracy of 0.959, a precision of 0.923, a recall of 1.000, an F1 score of 0.960, and an AUC of 0.945. Default values were used for all other parameters.

**Figure 6:**
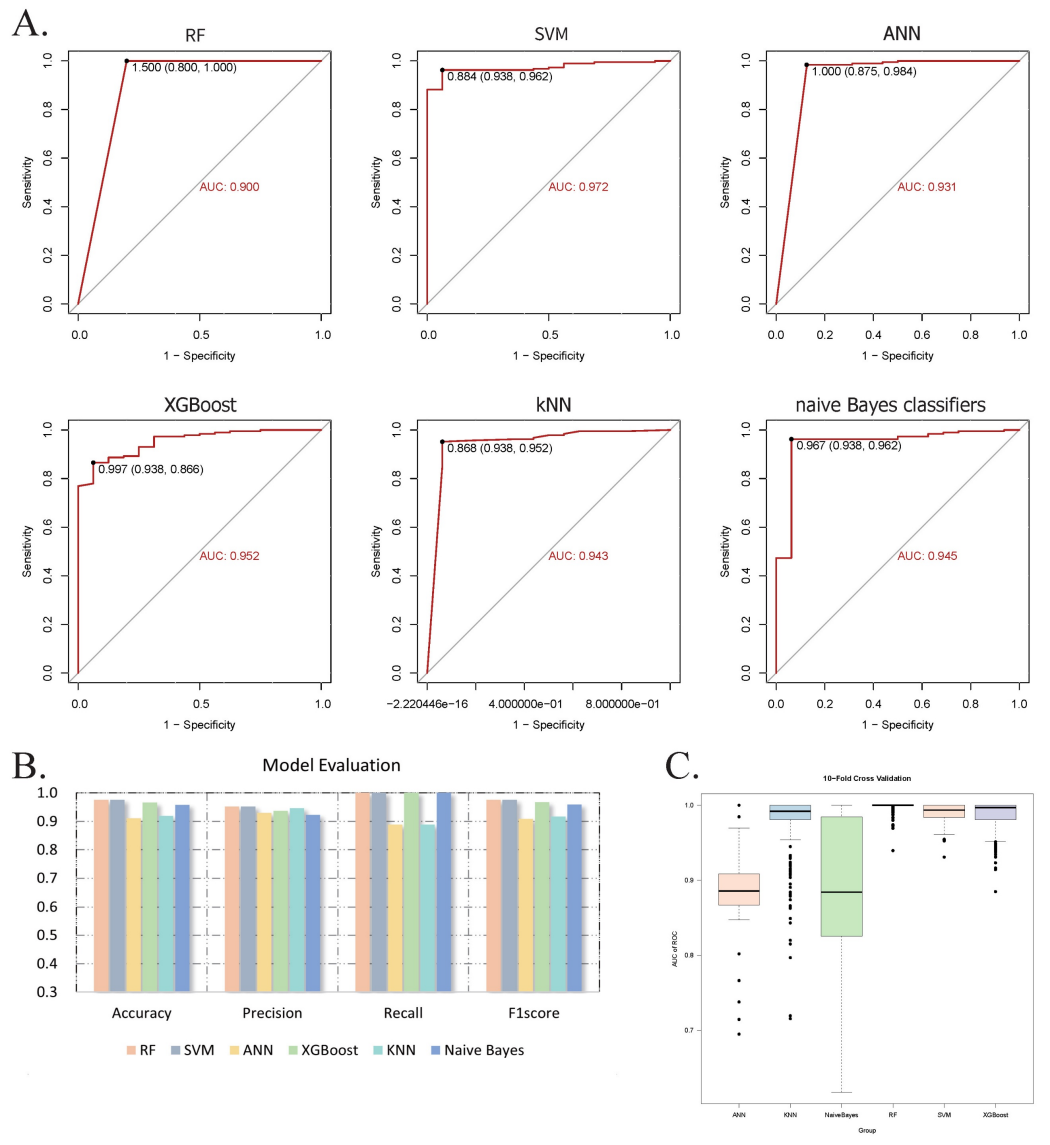
Evaluation of machine learning models. (A) ROC curves with corresponding AUC values for six classifiers: RF, SVM, ANN, XGBoost, kNN, and Naive Bayes. (B) Bar graph showing the accuracy, precision, recall, and F1 score of each model. (C) Box plot illustrating the distribution of AUC values from 400 iterations of 10-fold cross-validation, indicating model robustness. Abbreviations: ROC, receiver operator characteristic curve; AUC, area under the curve; RF, random forest; SVM, supporting vector machine; ANN, artificial neural network; XGBoost, extreme gradient boosting; kNN, k-nearest neighbor.

Model performance was visualized using ROC curves (Figure 6A-B), demonstrating consistently high discriminative ability across all six algorithms. To evaluate model robustness, we conducted 400 iterations of 10-fold cross-validation and displayed the AUC distributions using boxplots. The average AUCs were: 0.999 (RF), 0.991 (SVM), 0.891 (ANN), 0.988 (XGBoost), 0.980 (kNN), and 0.876 (Naive Bayes) (Figure 6C). Based on these results, a soft voting ensemble approach was employed, combining the probabilistic outputs of all models through weighted averaging to further enhance predictive accuracy and stability. The outcomes and model performances are provided in Figures 6 and S1.

### Single-cell sequencing analysis

**Figure 7:**
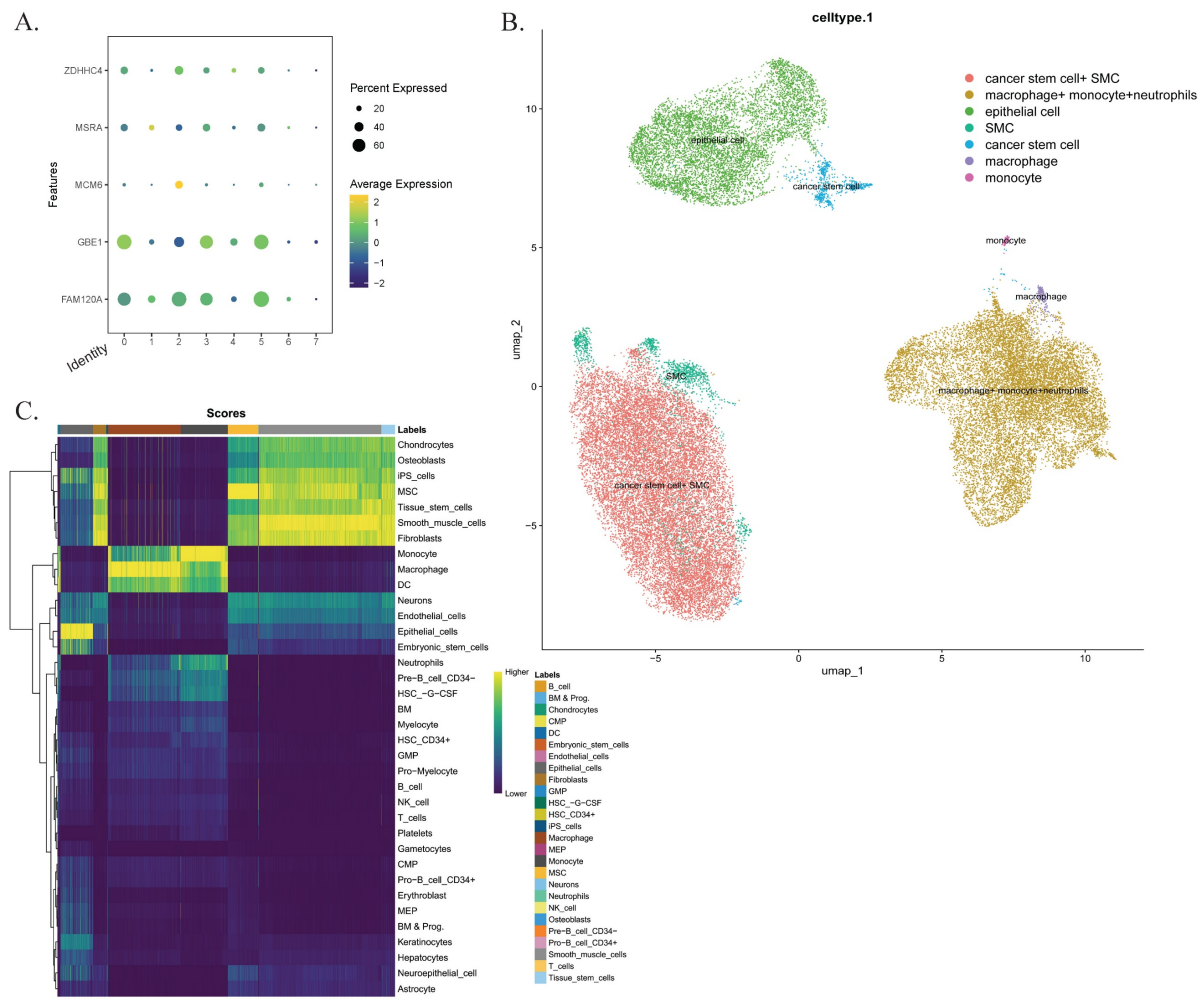
Single-cell sequencing analysis. (A) The characteristic atlas of cell subsets constructed based on the standardized gene expression matrix shows the expression levels of key marker genes in the cell subsets defined by the seven transcriptomes. (B) The nonlinear dimensionality reduction visualization results of UMAP show the single-cell clustering distribution. Cells are color-coded according to predefined subgroups. (C) Confidence interval heat maps, with light colors for higher confidence and dark colors for lower confidence. Abbreviations: UMAP, Uniform Manifold Approximation and Projection.

To investigate the infiltration of CD4^+^ T cells and M0 macrophages in CRC tissues, we analyzed a single-cell sequencing dataset in the GEO database, which included samples from 7 patients. After quality control, a total of 40,290 cells were included in the analysis. Through harmonized data integration, dimensionality reduction, and batch effect correction, we systematically annotated single-cell populations. We identified six cell types, including macrophages (PPP1R17), monocytes and neutrophils (FCAR, S100A12, CD93), tumor stem cells (CPZ), smooth muscle cell populations and epithelial cells (CSF3, TTC23L). These results were consistent with the findings from our previous analyses. Additionally, we examined the expression patterns of five key genes and their distribution within the tumor microenvironment, highlighting potential therapeutic targets (Figure 7).

## Discussion

CRC is one of the most prevalent malignancies worldwide, with its consistently poor prognosis largely attributed to delayed diagnosis and limited therapeutic options. Emerging evidence indicates that gut microbiota dysbiosis, in concert with host inflammatory responses, plays a critical role in CRC development. However, to our knowledge, no prior study has systematically investigated the mediating role of IBD in the causal pathway linking gut microbiota to CRC. To address this gap, we performed a mediation MR analysis and identified IBD as a significant mediator in the relationship between 12 gut microbial taxa and CRC, providing novel insights into the microbiota-inflammation-cancer axis. In addition, we identified five key genes as potential therapeutic targets. Transcriptomic analysis revealed dysregulated pathways involved in membrane-mediated signal transduction and cell cycle regulation. Immune infiltration analysis underscored the involvement of CD4⁺ T cells and M0 macrophages in CRC progression. A predictive model incorporating six machine learning algorithms demonstrated robust performance, and our results were further validated through single-cell sequencing analysis. These results enhance our understanding of CRC pathogenesis and provide promising implications for early diagnosis and targeted therapy.

Previous epidemiological studies and systematic reviews have consistently identified IBD as a significant risk factor for CRC. Chronic inflammation-induced oxidative stress is believed to contribute to CRC development by activating oncogenes and silencing tumor suppressor genes 7. Pharmacological interventions commonly used in IBD management—such as 5-aminosalicylic acid, thiopurine, nonsteroidal anti-inflammatory drugs, and tumor necrosis factor-α inhibitors—have been associated with reduced CRC risk 56. Our study provides supporting evidence for a causal link between the two diseases. To enhance robustness, we integrated multiple GWAS datasets and employed complementary MR methods. Although one MR analysis yielded an unexpectedly high OR, we retained the results based on the biological plausibility of the IBD-CRC association and the methodological strength of MR.

To further elucidate the role of gut microbiota in CRC pathogenesis, we conducted a two-sample MR analysis and identified 12 bacterial taxa with significant causal associations with both CRC and IBD. Several identified taxa—including *Bifidobacteriaceae*, *Eubacterium coprostanoligenes group*, *Bifidobacterium*, *RuminococcaceaeUCG011*, *Streptococcus*, an unknown genus (id.1868), and phylum* Actinobacteria*—are consistent with previous reports 57-59. A novel microbial marker (m3) from *Lachnoclostridium* has shown promise for non-invasive CRC detection 60. Additionally, members of the phylum and class *Actinobacteria*, such as *Tyzzerella nexilis*, have been associated with CRC development 61, while *Alcaligenaceae* has been reported to be enriched in the gut microbiota of CRC patients 62. Although *Peptococcus* has not been directly linked to CRC in previous studies, related genera such as *Peptostreptococcus* have been found to be enriched in both fecal and mucosal microbiota of CRC patients and implicated in tumor initiation and progression, suggesting a potential analogous role 63. Nevertheless, these observational findings cannot serve as definitive evidence for a direct relationship due to potential confounding factors, such as the environment and diet. Our MR analysis strengthens the evidence for causal relationships between specific gut microbial taxa and CRC risk.

Furthermore, mediation MR analysis revealed that IBD significantly mediates the relationship between these 12 taxa and CRC. Notably, IBD accounted for 55.73% of the total effect in the causal pathway from phylum *Actinobacteria* to CRC—the highest mediation proportion among all identified taxa. Prior studies suggest that certain *Actinobacteria* members exert both pro- and anti-inflammatory effects in IBD, which may influence CRC progression via immune modulation 66. These findings offer a mechanistic basis for the contribution of specific microbiota components to CRC in the context of intestinal inflammation 64.

To explore potential downstream molecular mediators, we mapped SNPs associated with these 12 microbial taxa and performed Wilcoxon rank-sum tests, identifying 37 candidate genes. LASSO regression was applied to refine these candidates, ultimately highlighting eight genes potentially involved in CRC pathogenesis. Subsequent drug-target MR and SMR analyses, based on eQTL data for seven of these genes, confirmed five genes—FAM120A, GBE1, MCM6, MSRA, and ZDHHC4—as significantly associated with CRC, supporting their potential as therapeutic targets and underscoring the robustness of our gene selection strategy.

The five identified genes demonstrate distinct roles in cancer biology. FAM120A promotes tumorigenesis via its circular RNA variant (circFAM120A), which enhances translation of the parental gene 65. GBE1, while traditionally linked to glycogen metabolism, has emerged as a tumor progression factor through its involvement in NF-κB-mediated FBP1 methylation in lung cancer 66. MCM6 encodes a conserved protein, which drives S/G2 phase cell cycle progression and serves as a potential diagnostic and prognostic biomarker in hepatocellular carcinoma. Notably, elevated MCM6 expression correlates with poor survival in gastric cancer patients 67. MSRA, a key enzyme in redox homeostasis, suppresses metastasis in pancreatic ductal adenocarcinoma via the MSRA-PKM2 axis, linking oxidative stress regulation to cancer metabolism 68. ZDHHC4 encodes a palmitoyltransferase that promotes the palmitoylation and membrane raft localization of KAI1, thereby enhancing its stability and anti-angiogenic function 69. In our study, the effects of these genes on CRC risk varied between the drug target MR and SMR analyses, with some genes exhibiting ORs less than 1 and others greater than 1. These discrepancies may reflect the complex and context-dependent roles of these genes in CRC pathogenesis. In addition, differences in data sources and analytical frameworks between the two methods could also contribute to the inconsistent results. Such variation underscores the need for further validation using more approaches and refined functional studies.

To elucidate the molecular mechanisms driving colorectal carcinogenesis, we integrated DEG analysis with WGCNA, identifying 1,666 genes for transcriptomic evaluation. GO enrichment analysis revealed significant involvement in the regulation of membrane potential, synaptic signaling, and muscular system processes. KEGG pathway analysis showed prominent enrichment in the neuroactive ligand-receptor interaction pathway. GSEA highlighted activation of key oncogenic pathways, particularly the G2/M checkpoint and apoptosis pathways. Current studies have underscored the critical role of the Wnt membrane-mediated signaling pathway in the development and progression of CRC, primarily through the regulation of key oncogenes such as c-Myc and cyclin D1. In addition, the PI3K-Akt signaling pathway is notably activated in CRC and promotes cell cycle progression from the G2 to M phase by regulating cell cycle regulators, including cyclin B1, cdc25C, and cdc2 70. The consistency between our findings and established CRC-related mechanisms supports the robustness and biological relevance of our analysis.

Immune infiltration analysis revealed relatively higher levels of plasma cells and mast cells in the low-LASSO score group. Plasma cells, as antibody-secreting effector cells, are known to enhance anti-tumor immune responses. The role of mast cells in CRC, however, remains controversial. While some studies have associated their infiltration with improved prognosis, others have reported adverse outcomes or even reduced survival, possibly due to the high heterogeneity of tumor microenvironments 71. Our results suggest that mast cells may exert anti-tumor effects in CRC, warranting further investigation into their functional contributions to tumor immunology. In contrast, the high-LASSO score group showed elevated levels of CD4⁺ T cells and M0 macrophages. CD4⁺ T cells can differentiate into various subsets, including Th1, Th2, Th17, and regulatory T cells (Tregs), each modulating the CRC immune microenvironment through specific cytokine profiles 72. Notably, increased infiltration of FOXP3⁺ Tregs has been linked to greater tumor aggressiveness and lymph node metastasis, primarily through their secretion of the immunosuppressive cytokine IL-10 73. M0 macrophages, as undifferentiated precursors, can polarize into M1 (pro-inflammatory) or M2 (anti-inflammatory) phenotypes in response to microenvironmental signals. The predominance of M2 macrophages in CRC is consistently associated with immunosuppression and tumor progression 74. Importantly, the gut microbiota modulates the function and differentiation of CD4⁺ T cells and macrophages through microbial-derived metabolites and immune signaling. For instance, short-chain fatty acids derived from microbial fermentation promote Tregs differentiation and function, while microbially modified bile acids modulate immune responses by affecting macrophage polarization 75. These findings collectively highlight the intricate interplay between gut microbiota and host immune responses in CRC pathogenesis.

Machine learning has become a widely adopted approach in biomedical informatics due to its ability to efficiently integrate and analyze complex datasets 76. In this study, we constructed six predictive models for CRC based on five key genes identified in previous analyses. Among these, the SVM model demonstrated the highest performance, with an AUC of 0.972, demonstrating promising potential for clinical application in CRC diagnosis and risk stratification. The integrated soft voting approach further enhanced predictive accuracy.

To verify gene expression at the cellular level, we used data from untreated CRC patients to ensure a reliable cell source. Key subpopulations included tumor stem cells, macrophages, monocytes, and intestinal epithelial cells, all of which are critical components in shaping the tumor microenvironment. These findings are consistent with previous studies. For example, single-cell sequencing analysis of CAFs and their role in extracellular matrix remodeling and angiogenesis, as reported by Hu *et al.*, demonstrated that CAFs in right-sided colon cancer exhibit a stronger cancer invasion signal. Additionally, the reprogramming of CD8^+^ T cells and macrophages plays a significant role in the malignant progression of CRC 77. Moreover, a significant causal relationship between various immune cell phenotypes—such as B cells, CD8^+^ T cells, Tregs, and monocytes—and CRC development has been reported 78. Xu *et al.*'s study also highlighted that the CD3 immune cell phenotype on CD28+CD4-CD8-T cells and the HLA-DR expression on CD33-HLA-DR+ cells exhibit protective effects against breast cancer 79. We also identified new subpopulations, such as smooth muscle cells, which have not been extensively described in previous studies, providing novel directions for CRC research. However, limitations such as sample size and sensitivity in detecting rare cell populations may influence the interpretation of these findings. Notably, FAM120A and GBE1 were significantly expressed in multiple cell types, suggesting their roles in CRC cell proliferation and providing new insights into their functions in tissue regeneration, inflammatory response, and tumor microenvironment regulation.

Our findings offer significant translational relevance and provide several directions for future research. By elucidating the mediating role of IBD in the causal relationship between gut microbiota and CRC, this study proposes a new mechanistic framework for understanding microbiota- and inflammation-driven carcinogenesis. Moreover, by integrating microbiome features, SNP mapping, and transcriptomic analyses, we identified candidate microbial and genetic biomarkers as well as functionally relevant pathways, which not only align with current studies but also offer novel opportunities for early detection and therapeutic targeting. The machine learning model developed in this study exhibited strong predictive performance, underscoring its potential utility in CRC risk stratification, particularly for high-risk IBD populations. As the field progresses, incorporating these markers into existing clinical workflows may enhance their sensitivity and specificity of early CRC screening. For instance, the fecal immunochemical testing-based prediction model proposed by Cubiella *et al.* has demonstrated robust diagnostic performance in symptomatic patients, highlighting the feasibility of biomarker-driven clinical applications 80. Similarly, the work of Díez Alonso *et al.* 81, which proposed incorporating tumor deposits into the definition of the TNM system contributes to the prognostic stratification of CRC. Building on these precedents, our study contributes additional value by introducing microbiome- and gene-based predictors. Future studies should validate these biomarkers and predictive models in large-scale, multicenter, and multi-ethnic prospective cohorts and evaluate their integration into multi-omics-driven screening and surveillance frameworks. These efforts hold promise for enabling more personalized and effective prevention and therapeutic strategies for inflammation-associated CRC.

The main strengths of this study include: 1) Comprehensive integration of dataset from IIBDGC, FinnGen, eQTLGen, GEO, and the published literature, which enhances the generalizability and robustness of the findings; 2) Application of diverse analytical methodologies, enabling multidimensional validation and improved analytical rigor; and 3) Cross-database validation, ensuring reproducibility and reliability of the results across different populations and study settings. However, several limitations should be acknowledged: 1) Although the genetic data were derived from multiple cohorts, most participants were of European and Asian ancestry. Future studies should include more ethnically diverse populations to improve the generalizability of the findings; 2) The single-factor MR analysis was based on the assumption of a linear relationship among gut microbiota, IBD, and CRC. To better capture complex biological interactions, future studies using individual-level data are needed to explore potential nonlinearities and interaction effects across different exposures. 3) The lack of eQTL data for certain genes limited comprehensive functional characterization. Incorporating additional multi-omics datasets in future research may provide deeper mechanistic insights; 4) While the identified genes and predictive models demonstrate strong potential, their clinical relevance requires further experimental and clinical investigations; 5) The focus on genetic determinants may restrict the model's ability to fully account for the multifactorial nature of CRC. Future work should incorporate data on environmental exposures and lifestyle factors to enhance explanatory power and clinical applicability.

## Conclusions

This study integrates MR, transcriptomic analysis, and machine learning to clarify the causal relationship between specific gut microbiota and CRC, and is the first to establish the mediating role of IBD in this pathway. These findings offer new mechanistic insights into the microbiota-inflammation-cancer axis and identify microbial and genetic markers, along with predictive models, with strong potential for early CRC detection and targeted interventions. In addition, immune infiltration and pathway enrichment analyses highlight key immunological and molecular features that may guide future therapeutic strategies. Collectively, our results lay a foundation for incorporating multi-omics biomarkers into clinical risk assessment for IBD-associated CRC. Future studies should focus on large-scale clinical validation and functional experiments to facilitate the translation of these findings into effective prevention and treatment strategies.

## Supplementary Material

Supplementary tables 1-11, figure.

Supplementary tables 2-20.

## Figures and Tables

**Table 1:** Mendelian randomization analyses between gut microbiota and two CRC datasets of inverse variance weighted method.

Trait	IVW-OR (95%CI, P-value)	
	Colorectal cancer (Huyghe JR *et al.*)	Colorectal cancer (Ishigaki K *et al.*)
class.Actinobacteria.id.419	0.843(0.810-0.876, 0)	0.903(0.876-0.930, 0)
family.Alcaligenaceae.id.2875	0.504(0.439-0.569, 0)	1.508(1.389-1.636, 0)
family.Bifidobacteriaceae.id.433	0.889(0.854-0.925, 0)	0.912(0.891-0.937, 0)
genus.Eubacteriumcoprostanoligenesgroup.id.11375	1.634(1.358-1.910, 0)	1.222(1.118-1.336, 0)
genus.Bifidobacterium.id.436	0.864(0.831-0.898, 0)	0.888(0.865-0.912, 0)
genus.Lachnoclostridium.id.11308	0.604(0.518-0.704, 0)	0.809(0.725-0.901, 0)
genus.Peptococcus.id.2037	0.924(0.882-0.969, 0.001)	1.262(1.224-1.300, 0)
genus.RuminococcaceaeUCG011.id.11368	1.371(1.314-1.430, 0)	1.249(1.203-1.297, 0)
genus.Streptococcus.id.1853	0.415(0.384-0.449, 0)	0.845(0.745-0.959, 0.009)
genus.unknowngenus.id.1868	0.875(0.804-0.952, 0.002)	1.082(1.027-1.141, 0.003)
order.Bifidobacteriales.id.432	0.889(0.854-0.925, 0)	0.912(0.891-0.937, 0)
phylum.Actinobacteria.id.400	0.844(0.791-0.901, 0)	0.913(0.879-0.948, 0)

IVW, inverse variance weighted method; OR, odds ratio; CI, confidential interval; a P-value of 0 means the P-value < 0.001.

**Table 2:** Mendelian randomization analyses between gut microbiota and three IBD datasets of inverse variance weighted method.

Trait	IVW-OR (95%CI, P-value)		
	IBD (FinnGen)	IBD (IIBDGC)	IBD (Mbatchou J *et al.*)
class.Actinobacteria.id.419	0.830(0.810-0.851, 0)	0.805(0.788-0.823, 0)	0.997(0.996-0.997, 0)
family.Alcaligenaceae.id.2875	1.279(1.185-1.381, 0)	0.887(0.832-0.946, 0)	0.999(0.998-0.999, 0)
family.Bifidobacteriaceae.id.433	0.782(0.761-0.803, 0)	0.784(0.766-0.802, 0)	0.996(0.996-0.997, 0)
genus.Eubacteriumcoprostanoligenesgroup.id.11375	1.161(1.027-1.313, 0.017)	1.320(1.201-1.451, 0)	1.006(1.005-1.007, 0)
genus.Bifidobacterium.id.436	0.789(0.768-0.811, 0)	0.806(0.788-0.824, 0)	0.997(0.996-0.997, 0)
genus.Lachnoclostridium.id.11308	0.507(0.460-0.560, 0)	0.885(0.816-0.960, 0.003)	1.002(1.001-1.003, 0)
genus.Peptococcus.id.2037	0.912(0.885-0.939, 0)	1.166(1.137-1.195, 0)	0.999(0.999-1.000, 0)
genus.RuminococcaceaeUCG011.id.11368	1.071(1.044-1.098, 0)	1.043(1.020-1.067, 0)	1.001(1.001-1.001, 0)
genus.Streptococcus.id.1853	0.795(0.752-0.841, 0)	1.417(1.359-1.477, 0)	1.003(1.002-1.003, 0)
genus.unknowngenus.id.1868	0.881(0.822-0.944, 0)	1.069(1.022-1.119, 0.004)	1.001(1.000-1.001, 0.004)
order.Bifidobacteriales.id.432	0.782(0.761-0.803, 0)	0.784(0.766-0.802, 0)	0.996(0.996-0.997, 0)
phylum.Actinobacteria.id.400	0.765(0.734-0.797, 0)	0.721(0.695-0.748, 0)	0.995(0.995-0.996, 0)

IVW, inverse variance weighted method; OR, odds ratio; CI, confidential interval; a P-value of 0 means the P-value < 0.001.

**Table 3:** Mediation analysis of the effect of gut microbiota on CRC via IBD.

Exposure	Mediator	Outcome	Total effect	Direct effect	Mediation effect	Mediation proportion (%)
family.Alcaligenaceae.id.2875	IBD (FinnGen)	CRC (Huyghe JR *et al.*)	-0.693	-0.667	-0.026	3.800%
genus.Streptococcus.id.1853	IBD (IIBDGC)	CRC (Huyghe JR *et al.*)	-0.879	-0.858	-0.021	2.440%
genus.unknowngenus.id.1868	IBD (IIBDGC)	CRC (Huyghe JR *et al.*)	-0.133	-0.129	-0.004	3.082%
genus.Peptococcus.id.2037	IBD (IIBDGC)	CRC (Huyghe JR *et al.*)	-0.079	-0.069	-0.009	12.007%
class.Actinobacteria.id.419	IBD (Mbatchou J *et al.*)	CRC (Ishigaki K *et al.*)	-0.103	-0.065	-0.038	36.997%
family.Bifidobacteriaceae.id.433	IBD (Mbatchou J *et al.*)	CRC (Ishigaki K *et al.*)	-0.090	-0.049	-0.041	45.678%
genus.Eubacteriumcoprostanoligenesgroup.id.11375	IBD (Mbatchou J *et al.*)	CRC (Ishigaki K *et al.*)	0.200	0.137	0.064	31.848%
genus.Bifidobacterium.id.436	IBD (Mbatchou J *et al.*)	CRC (Ishigaki K *et al.*)	-0.119	-0.082	-0.037	30.857%
genus.RuminococcaceaeUCG011.id.11368	IBD (Mbatchou J *et al.*)	CRC (Ishigaki K *et al.*)	0.222	0.209	0.014	6.129%
genus.unknowngenus.id.1868	IBD (Mbatchou J *et al.*)	CRC (Ishigaki K *et al.*)	0.079	0.071	0.008	10.232%
order.Bifidobacteriales.id.432	IBD (Mbatchou J *et al.*)	CRC (Ishigaki K *et al.*)	-0.090	-0.049	-0.041	45.678%
phylum.Actinobacteria.id.400	IBD (Mbatchou J *et al.*)	CRC (Ishigaki K *et al.*)	-0.091	-0.040	-0.051	55.725%

**Table 4:** Drug target Mendelian randomization and SMR results.

Gene	IVW-OR (95%CI, P-value)		SMR-OR (95%CI)	P-SMR	P-HEIDI	Number of Significant SNPs in SMR
	Colorectal cancer (Huyghe JR *et al.*)	Colorectal cancer (Ishigaki K *et al.*)				
ADCYAP1R1	N/A					
ATP11A	1.361(1.173-1.580, 0)	0.945(0.855-1.044, 0.263)	1.240(0.715-2.148)	0.444	0.592	0
FAM120A	0.752(0.716-0.791, 0)	0.841(0.813-0.869, 0)	1.283(0.990-1.663)	0.059	0.228	1
GBE1	1.082(1.052-1.113, 0)	0.929(0.912-0.946, 0)	0.950(0.738-1.223)	0.689	0.062	3
MCM6	0.969(0.963-0.975, 0)	0.969(0.965-0.972, 0)	1.241(1.017-1.516)	0.034	0.115	2
MSRA	0.941(0.935-0.947, 0)	1.030(1.025-1.035, 0)	0.846(0.718-0.997)	0.046	0.259	7
SOAT1	1.072(1.063-1.081, 0)	1.006(0.997-1.014, 0.180)	1.174(0.963-1.432)	0.113	0.500	0
ZDHHC4	0.950(0.939-0.961, 0)	1.003(0.992-1.014, 0.632)	0.887(0.815-0.965)	0.006	0.250	4

IVW, inverse variance weighted method; OR, odds ratio; CI, confidential interval; a P-value of 0 means the P-value<0.001.

## Abbreviations

MR: Mendelian randomization
CRC: colorectal cancer
IBD: inflammatory bowel disease
GWAS: genome-wide association studies
SNPs: single nucleotide polymorphisms
LASSO: least absolute shrinkage and selection operator
SMR: summary-data-based Mendelian randomization
IIBDGC: International Inflammatory Bowel Disease Genetics Consortium
eQTL: expression quantitative trait locus
TCGA: The Cancer Genome Atlas
GEO: gene expression omnibus
IVs: instrumental variables
IVW: inverse-variance weighted
HEIDI: heterogeneity in dependent instruments
WGCNA: weighted gene co-expression network analysis
DEG: differentially expressed gene
GO: gene ontology
KEGG: Kyoto Encyclopedia of genes and genomes
GSEA: gene set enrichment analysis
RF: random forest
SVM: support vector machine
XGBoost: extreme gradient boosting
ANN: artificial neural networks
kNN: k-nearest neighbors
AUC: area under the curve
ROC: receiver operating characteristic
OR: odds ratio
