# Supplementary material for: Inflammatory Bowel Disease Mediates the Causal Relationship Between Gut Microbiota and Colorectal Cancer: Identification of Therapeutic Targets and Predictive Modeling
Source: J Cancer. 2025 Sep 22;16(13):4008–28. doi: 10.7150/jca.114687 (PMC12491167; doi:10.7150/jca.114687)
Supplement: Supplementary file 1 — Supplementary tables 1-11, figure. [file jcav16p4008s1.pdf]

Supplementary File 1

Table S1: Detailed MR and sensitivity analyses between microbiota and inflammatory bowel disease using FinnGen GWAS data.

Table S2: Detailed MR and sensitivity analyses between microbiota and inflammatory bowel disease using IIBDGC GWAS data.

Table S3: Detailed MR and sensitivity analyses between microbiota and inflammatory bowel disease using Mbatchou J et al.'s GWAS data.

Table S4: Detailed MR analyses between inflammatory bowel disease and colorectal cancer.

Table S5: Detailed MR analyses between microbiota and colorectal cancer using Huyghe JR et al.'s GWAS data.

Table S6: Detailed MR analyses between microbiota and colorectal cancer using Ishigaki K et al.'s GWAS data.

Table S7: The Mendelian randomization results of the microbiota and IBD which had mediating effects in CRC.

Table S8: Expression analysis and LASSO regression.

Table S9: Detailed results of drug target MR analyses between seven candidate genes and colorectal cancer using Huyghe JR et al.'s GWAS data.

Table S10: Detailed results of drug target MR analyses between seven candidate genes and colorectal cancer using Ishigaki K et al.'s GWAS data.

Table S11: Detailed results of SMR and HEIDI tests between the expression of seven candidate genes and colorectal cancer using Ishigaki K et al.'s GWAS data.

Figure S1: Parameter tuning, variable importance, and model performance evaluation of six machine learning algorithms used for CRC classification.

Table S1:

Detailed MR and sensitivity analyses between microbiota and inflammatory bowel disease using FinnGen GWAS data.

Trait	OR(IVW)	95%CI(IVW)	Pval(IVW)	OR(MR-Egger)	95%CI(MR-Egger)	Pval(MR-Egger)	OR(Weighted median)	95%CI(Weighted median)	Pval(Weighted median)	OR(Weighted mode)	95%CI(Weighted mode)	Pval(Weighted mode)	OR(Simple mode)	95%CI(Simple mode)	Pval(Simple mode)	Rucker Q(MR-Egger)	Pval_Q(MR-Egger)	Cochran Q(IVW)	Pval_Q(IVW)	Intercept(MR-Egger)	Pval_Intercept	Power	Steiger P-value	Number of SNP Not Passing MR-Steiger	P-adj(IVW)
class.Actinobacteria.id.419	0.830114185	0.810-0.851	2.9865E-48	0.690994703	0.613-0.778	2.1619E-09	0.821630886	0.794-0.850	8.35922E-30	0.825231235	0.740-0.921	0.000612473	0.825231235	0.732-0.930	0.001704942	452.8329148	0.999958597	462.3627164	0.999863028	0.015741907	0.002118659	1	NA	0	1.575377E-46
family.Alcaligenaceae.id.2875	1.278980433	1.185-1.381	3.19309E-10	0.428524404	0.241-0.762	0.004938608	1.398965304	1.268-1.543	1.9569E-11	1.412117798	1.101-1.811	0.007858339	1.412117798	1.109-1.797	0.006180176	16.55133177	1	30.64004547	0.999999999	0.063476366	0.000310412	0.999699913974686	0	0	3.368705E-09
family.Bifidobacteriaceae.id.433	0.781641809	0.761-0.803	1.45307E-74	1.072143858	0.952-1.207	0.24962162	0.830182878	0.801-0.861	6.18111E-24	0.832590551	0.749-0.926	0.000778142	0.832590551	0.741-0.936	0.002275238	486.7639447	0.494495	515.4595642	0.188284115	-0.027322842	1.30855E-07	1	NA	0	1.532988E-72
genus.Eubacteriumcoprostanoligenesgroup.id.11375	1.161357197	1.027-1.313	0.016677897	0.679804855	0.305-1.516	0.350615793	1.198258154	1.021-1.406	0.026909628	1.236377439	0.908-1.684	0.184885066	1.242803009	0.907-1.704	0.183421353	10.03699249	0.999999991	11.79077906	0.999999926	0.041312018	0.192088528	0.961749067304915	5.21111284895923e-220	0	5.324683E-02
genus.Bifidobacterium.id.436	0.789139645	0.768-0.811	2.30783E-63	1.157571278	1.020-1.313	0.023277861	0.824332467	0.794-0.855	1.12287E-24	0.833493401	0.756-0.919	0.000272625	0.829938184	0.739-0.932	0.001805521	602.2684234	0.012652304	644.7474445	0.000365505	-0.032717356	2.09416E-09	1	NA	0	1.623176E-61
genus.Lachnoclostridium.id.11308	0.507598756	0.460-0.560	6.74382E-42	1.904478222	0.853-4.253	0.121203609	0.445089595	0.386-0.513	1.02072E-28	0.4162189	0.298-0.582	3.13225E-06	0.414909216	0.306-0.563	4.14962E-07	36.56443644	0.994487623	47.12808715	0.919110441	-0.072563762	0.001879085	0.99002993386255	5.25248431963958e-247	0	2.371577E-40
genus.LachnospiraceaeND3007group.id.11317	1.554014627	1.207-2.000	0.000622449	0.603650241	1.497-243475.231	0.941048678	1.546101809	1.129-2.117	0.00656180106899353	1.51357243607311	0.956-2.397	0.115178122932628	1.51357243607311	1.009-2.271	0.0801983243181022	0.0719936197477242	0.999999260174171	0.0926193252833435	0.999999815329275	0.0516021221675589	0.889850266363259	1.129-2.117	0.00656180106899353	0	2.626734E-03
genus.Peptococcus.id.2037	0.911836931	0.885-0.939	6.76529E-10	1.247928773	1.023-1.522	0.029934736	0.844706213	0.811-0.879	2.45206E-16	0.844772467	0.765-0.933	0.001045464	0.844772467	0.759-0.941	0.002418547	100.3725139	0.999999978	110.1914013	0.999999111	-0.036964485	0.002004123	1	0	0	6.797502E-09
genus.RuminococcaceaeUCG011.id.11368	1.070770632	1.044-1.098	1.39456E-07	0.33471752	0.266-0.420	3.54369E-17	1.145886928	1.106-1.187	3.82432E-14	1.146857159	1.033-1.274	0.01123225	1.146857159	1.035-1.270	0.009350782	14.87997836	1	115.9932305	0.999571781	0.144356661	5.84044E-19	1	NA	0	9.808399E-07
genus.Streptococcus.id.1853	0.795489014	0.752-0.841	9.0014E-16	0.548241022	0.427-0.703	4.8003E-06	0.788019677	0.733-0.848	1.55569E-10	0.789046231	0.643-0.968	0.024651704	0.789046231	0.634-0.982	0.03484855	38.03422663	1	47.06000117	1	0.04011826	0.003072089	0.999999999937151	0	0	1.726632E-14
genus.unknowngenus.id.1868	0.881000639	0.822-0.944	0.000316869	2.190787339	1.299-3.695	0.004014713	0.682185686	0.628-0.741	1.12599E-19	0.67447734	0.578-0.787	2.06769E-06	0.67312999	0.565-0.801	2.10204E-05	155.2711774	0.001967043	172.3073877	0.000106566	-0.072451005	0.000821479	0.999999990267841	0	0	1.364476E-03
order.Bifidobacteriales.id.432	0.781641809	0.761-0.803	1.45307E-74	1.072143858	0.952-1.207	0.24962162	0.830182878	0.801-0.861	6.46247E-24	0.832590551	0.757-0.916	0.000188936	0.832590551	0.744-0.931	0.001415213	486.7639447	0.494495	515.4595642	0.188284115	-0.027322842	1.30855E-07	1	NA	0	1.532988E-72
phylum.Actinobacteria.id.400	0.764931281	0.734-0.797	2.0622E-36	0.445225824	0.334-0.593	7.62367E-08	0.768167834	0.726-0.813	5.53746E-20	0.770290459	0.653-0.908	0.002126723	0.770290459	0.655-0.906	0.00174271	130.802303	1	144.762702	1	0.037917887	0.000227509	0.999999999999998	0	0	6.216051E-35

Abbreviation: OR, odds ratio; IVW, inverse variance weighted method; CI, confidential interval; a Pval of 0 means Pval<0.001; P-adj, P-value after P-value adjustment

Table S2:

Detailed MR and sensitivity analyses between microbiota and inflammatory bowel disease using IIBDGC GWAS data.

Trait	OR(IVW)	95%CI(IVW)	Pval(IVW)	OR(MR-Egger)	95%CI(MR-Egger)	Pval(MR-Egger)	OR(Weighted median)	95%CI(Weighted median)	Pval(Weighted median)	OR(Weighted mode)	95%CI(Weighted mode)	Pval(Weighted mode)	OR(Simple mode)	95%CI(Simple mode)	Pval(Simple mode)	Rucker Q(MR-Egger)	Pval_Q(MR-Egger)	Cochran Q(IVW)	Pval_Q(IVW)	Intercept(MR-Egger)	Pval_Intercept	Power	Steiger P-value	Number of SNP Not Passing MR-Steiger	P-adj(IVW)
class.Actinobacteria.id.419	0.805460076	0.788-0.823	2.48419E-82	0.497560277	0.447-0.554	9.40785E-33	0.855928113	0.828-0.885	3.82273E-20	0.903719169	0.818-0.998	0.046355982	0.622685824	0.547-0.709	2.98049E-12	431.576586	0.999999816	511.4799875	0.991937341	0.041015844	5.01816E-18	1	NA	0	1.308200E-80
family.Alcaligenaceae.id.2875	0.886988674	0.832-0.946	0.000272588	1.109917885	0.711-1.734	0.64790858	0.880934426	0.809-0.960	0.003664272	0.886004929	0.735-1.069	0.208870415	0.886004929	0.723-1.085	0.245091555	25.49217104	1	26.48347508	1	-0.013031274	0.32212317	0.999999812965996	1.66209684158975e-291	0	1.437902E-03
family.Bifidobacteriaceae.id.433	0.783964512	0.766-0.802	6.75641E-99	0.669620278	0.603-0.744	2.83944E-13	0.831250397	0.804-0.859	5.98714E-28	0.902923802	0.804-1.014	0.085008356	0.643811418	0.562-0.738	4.86619E-10	312.5265726	1	321.6561534	1	0.013605627	0.002644215	1	NA	0	7.131800E-97
genus.Eubacteriumcoprostanoligenesgroup.id.11375	1.319727304	1.201-1.451	9.07258E-09	2.114084009	1.081-4.135	0.033968068	1.402057008	1.229-1.599	4.78967E-07	1.44515067	1.124-1.858	0.006111729	1.448892628	1.101-1.906	0.010986265	16.93839991	0.999954493	18.87199879	0.999868145	-0.036399725	0.171204373	0.998208638322675	5.3069998132714e-124	0	8.320739E-08
genus.Bifidobacterium.id.436	0.805665752	0.788-0.824	1.04127E-82	0.645639334	0.581-0.718	3.38496E-15	0.853074961	0.826-0.881	3.37519E-22	0.903766716	0.814-1.003	0.05811452	0.628498666	0.551-0.716	9.59778E-12	325.371617	1	342.9882854	1	0.018823437	3.16033E-05	1	NA	0	7.314667E-81
genus.Lachnoclostridium.id.11308	0.885260553	0.816-0.960	0.003312507	1.394571881	0.598-3.251	0.444188851	0.882315545	0.793-0.981	0.021076063	0.881745793	0.705-1.102	0.273104911	0.880119035	0.692-1.119	0.302132179	8.348884467	1	9.466891684	1	-0.024554417	0.294586594	0.999840777522633	6.25701442638981e-187	0	1.270798E-02
genus.LachnospiraceaeND3007group.id.11317	1.526049397	1.209-1.927	0.000378189	0.614930502	0.005-82.010	0.850426075	1.515877194	1.145-2.007	0.003644433	1.510862786	0.984-2.320	0.092030841	1.511929559	0.977-2.339	0.09632106	0.044705495	0.99999999	0.177564563	0.999999671	0.050339363	0.724932556	0.650324470218474	1.55197931133598e-25	0	1.946290E-03
genus.Peptococcus.id.2037	1.165715926	1.137-1.195	1.79085E-33	0.816350485	0.689-0.967	0.020197589	1.191416239	1.153-1.231	1.08259E-25	1.181592651	1.074-1.300	0.000717025	1.181592651	1.079-1.294	0.00042621	35.02898291	1	52.30890766	1	0.041775845	4.84675E-05	1	0	0	4.196556E-32
genus.RuminococcaceaeUCG011.id.11368	1.043095251	1.020-1.067	0.000197942	0.752471304	0.610-0.928	0.008655226	1.06098418	1.031-1.092	5.37026E-05	1.079748642	0.997-1.170	0.061780275	1.079748642	0.994-1.172	0.069239327	15.80760814	1	25.20837234	1	0.040293387	0.002509171	1	NA	0	1.099099E-03
genus.Streptococcus.id.1853	1.416790672	1.359-1.477	1.37564E-59	2.017181004	1.683-2.418	1.92109E-12	1.514926649	1.431-1.604	5.53529E-46	1.584509684	1.364-1.841	1.03892E-08	1.590147471	1.369-1.847	7.66711E-09	118.4927094	0.999209562	133.9449339	0.985723134	-0.038228336	0.00012271	1	0	0	4.853000E-58
genus.unknowngenus.id.1868	1.0690961	1.022-1.119	0.003808806	1.334135717	0.931-1.911	0.118772565	1.216884457	1.142-1.297	1.49253E-09	1.266486479	1.049-1.529	0.015636919	1.269815003	1.064-1.515	0.009075242	97.83456659	0.844454952	99.31630786	0.834720685	-0.017709114	0.226038302	1	0	0	1.339430E-02
order.Bifidobacteriales.id.432	0.783964512	0.766-0.802	6.75641E-99	0.669620278	0.603-0.744	2.83944E-13	0.831250397	0.805-0.858	6.1979E-30	0.902923802	0.803-1.015	0.086923184	0.643811418	0.564-0.734	1.33566E-10	312.5265726	1	321.6561534	1	0.013605627	0.002644215	1	NA	0	7.131800E-97
phylum.Actinobacteria.id.400	0.720972989	0.695-0.748	1.62935E-68	0.59690824	0.465-0.766	6.77103E-05	0.778984303	0.738-0.822	1.03807E-19	0.867422029	0.742-1.014	0.076056653	0.867422029	0.730-1.030	0.10599125	178.4730745	0.999999671	180.7124472	0.999999497	0.013106682	0.135657861	1	0	0	6.878600E-67

Abbreviation: OR, odds ratio; IVW, inverse variance weighted method; CI, confidential interval; a Pval of 0 means Pval<0.001; P-adj, P-value after P-value adjustment

Table S3:

Detailed MR and sensitivity analyses between microbiota and inflammatory bowel disease using Mbatchou J et al.'s GWAS data.

Trait	OR(IVW)	95%CI(IVW)	Pval(IVW)	OR(MR-Egger)	95%CI(MR-Egger)	Pval(MR-Egger)	OR(Weighted median)	95%CI(Weighted median)	Pval(Weighted median)	OR(Weighted mode)	95%CI(Weighted mode)	Pval(Weighted mode)	OR(Simple mode)	95%CI(Simple mode)	Pval(Simple mode)	Rucker Q(MR-Egger)	Pval_Q(MR-Egger)	Cochran Q(IVW)	Pval_Q(IVW)	Intercept(MR-Egger)	Pval_Intercept	Power	Steiger P-value	Number of SNP Not Passing MR-Steiger	P-adj(IVW)
class.Actinobacteria.id.419	0.996544241	0.996-0.997	1.7847E-117	9.95E-01	0.993-0.996	3.36083E-14	9.97E-01	0.997-0.997	6.3418E-48	0.99750545	0.996-0.999	1.9271E-05	9.97E-01	0.996-0.999	0.00040252	767.6862441	2.63889E-06	778.7814063	8.07393E-07	0.000171535	0.003437502	1	NA	0	1.249276E-115
family.Alcaligenaceae.id.2875	0.99863805	0.998-0.999	0.000205467	0.993899525	0.989-0.999	0.018056152	0.998944529	0.998-1.000	0.035730913	0.998964631	0.997-1.001	0.351864206	0.998964631	0.997-1.001	0.353616609	56.88363547	0.997506653	60.46308573	0.99431191	0.000276912	0.061713742	0.996170574438701	0	0	9.922914E-04
family.Bifidobacteriaceae.id.433	0.996247737	0.996-0.997	1.097E-155	9.94E-01	0.993-0.995	5.37942E-20	9.97E-01	0.997-0.997	1.91502E-48	0.997824043	0.997-0.999	0.00051575	9.98E-01	0.996-0.999	0.004844426	535.7794105	0.129979011	550.1502798	0.063560071	0.000191423	0.000276789	1	NA	0	1.151881E-153
genus.Eubacteriumcoprostanoligenesgroup.id.11375	1.005838873	1.005-1.007	5.57818E-26	1.00798246	1.000-1.016	0.055628757	1.01E+00	1.006-1.009	2.92683E-21	1.007703786	1.005-1.011	1.40284E-05	1.007738071	1.005-1.011	3.82069E-06	52.20530819	0.245480599	52.52509659	0.268571422	-0.00016465	0.598092424	0.896066305134508	3.53412455182894e-217	0	1.301574E-24
genus.Bifidobacterium.id.436	0.996660004	0.996-0.997	4.6159E-110	9.96E-01	0.994-0.997	2.01505E-10	9.97E-01	0.997-0.998	8.71976E-48	0.997806247	0.997-0.999	0.000324698	9.96E-01	0.994-0.997	1.50096E-08	714.5127389	1.29166E-06	718.323652	9.26085E-07	9.73468E-05	0.088776455	1	NA	0	2.423351E-108
genus.Lachnoclostridium.id.11308	1.002007232	1.001-1.003	1.85252E-05	0.995353928	0.986-1.005	0.349590457	1.002061157	1.001-1.003	0.000439785	1.002372513	1.000-1.005	0.05879437	1.002372513	1.000-1.005	0.068778765	9.140893504	1	10.97684102	1	0.000359454	0.180592166	0.950070006006836	2.73773018312391e-297	0	1.080639E-04
genus.LachnospiraceaeND3007group.id.11317	1.002530758	1.000-1.005	0.0213831	0.976604603	0.932-1.023	0.348626907	1.002852063	1.000-1.006	0.035161468	1.002855505	0.999-1.007	0.202134909	1.002855505	0.999-1.007	0.19286303	0.165302338	0.99999818	1.381938708	0.997931518	0.001449449	0.302093655	0.33523523076676	7.10006190385631e-46	0	5.756989E-02
genus.Peptococcus.id.2037	0.999327556	0.999-1.000	1.83108E-06	1.001288975	0.999-1.003	0.173456107	9.99E-01	0.999-0.999	4.66262E-07	0.999119471	0.998-1.000	0.102496887	0.999119471	0.998-1.000	0.102890052	32.81177968	1	37.23524373	1	-0.000230501	0.036724063	1	0	0	1.13096E-05
genus.RuminococcaceaeUCG011.id.11368	1.001244635	1.001-1.001	2.06004E-22	1.00923661	1.007-1.012	1.70717E-12	1.00E+00	1.000-1.001	5.08742E-06	1.000681988	1.000-1.002	0.166676605	1.000681988	1.000-1.002	0.162917032	34.0023509	1	77.58464008	1	-0.000980408	4.5148E-10	1	NA	0	4.326084E-21
genus.Streptococcus.id.1853	1.002538852	1.002-1.003	4.33492E-26	1.01E+00	1.007-1.011	3.39594E-16	1.00E+00	1.001-1.002	0.000145365	1.00E+00	0.999-1.003	0.374424112	1.00E+00	0.999-1.003	0.3857454	74.69525957	1	120.3876916	0.999437444	-0.000734477	2.00889E-10	0.999999953698397	0	0	1.137916E-24
genus.unknowngenus.id.1868	1.000738489	1.000-1.001	0.003952954	1.002969547	0.999-1.007	0.115100563	1.00E+00	1.001-1.003	3.14618E-07	1.001955925	1.000-1.004	0.042870672	1.001955925	1.000-1.004	0.042871632	88.98489531	0.960040829	90.43421263	0.956114038	-0.000179307	0.231131314	0.999998545866264	0	0	1.431242E-02
order.Bifidobacteriales.id.432	0.996247737	0.996-0.997	1.097E-155	9.94E-01	0.993-0.995	5.37942E-20	9.97E-01	0.997-0.997	8.40591E-46	0.997824043	0.997-0.999	0.000330908	9.98E-01	0.996-0.999	0.006670869	535.7794105	0.129979011	550.1502798	0.063560071	0.000191423	0.000276789	1	NA	0	1.151881E-153
phylum.Actinobacteria.id.400	0.995366467	0.995-0.996	1.3E-82	0.992394503	0.989-0.996	0.00000278	0.996994512	0.996-0.998	1.85E-21	0.997450582	0.996-0.999	0.00058597	0.997354455	0.996-0.999	0.001442321	338.1872456	0.01091321	342.5099316	0.007907117	0.000206869	0.059094266	0.999999999966559	0	0	5.470782E-81

Abbreviation: OR, odds ratio; IVW, inverse variance weighted method; CI, confidential interval; a Pval of 0 means Pval<0.001; P-adj, P-value after P-value adjustment

Table S4:

Detailed MR analyses between inflammatory bowel disease and colorectal cancer.

Mediator	Outcome	Method	Number.of.SNPs	Beta	Se	Pval	LO_CI	UP_CI	OR	OR_LCI95	OR_UCI95	Rucker Q(MR-Egger)	Pval_Q(MR-Egger)	Cochran Q(IVW)	Pval_Q(IVW)	Intercept(MR-Egger)	Pval_Intercept	Power	Steiger P-value	Number of SNP Not Passing MR-Steiger
Inflammatory bowel disease (FinnGen)	Colorectal cancer (Huyghe JR et al.)	MR Egger	37	0.021505427	0.123301312	0.862545406	-0.220165145	0.263175998	1.021738335	0.802386277	1.301055682	22.51420319	0.949177135	23.80436596	0.940706457	-0.020465476	0.263736119			
Inflammatory bowel disease (FinnGen)	Colorectal cancer (Huyghe JR et al.)	Inverse variance weighted	37	-0.10710114	0.048820055	0.028250229	-0.202788448	-0.011413832	0.898434801	0.816450945	0.988651059	22.51420319	0.949177135	23.80436596	0.940706457	-0.020465476	0.263736119			
Inflammatory bowel disease (FinnGen)	Colorectal cancer (Huyghe JR et al.)	Weighted median	37	-0.097991552	0.067134831	0.144393677	-0.22957582	0.033592716	0.906656563	0.794870699	1.034163323	22.51420319	0.949177135	23.80436596	0.940706457	-0.020465476	0.263736119	0.551262560457053	0.263025826646883	9
Inflammatory bowel disease (FinnGen)	Colorectal cancer (Huyghe JR et al.)	Weighted mode	37	-0.104554947	0.113142721	0.361588329	-0.326314679	0.117204786	0.900725304	0.721578086	1.124349657	22.51420319	0.949177135	23.80436596	0.940706457	-0.020465476	0.263736119			
Inflammatory bowel disease (FinnGen)	Colorectal cancer (Huyghe JR et al.)	Simple mode	37	-0.093196526	0.132234134	0.485479544	-0.352375429	0.165982377	0.911014444	0.70301614	1.180552296	22.51420319	0.949177135	23.80436596	0.940706457	-0.020465476	0.263736119			

Mediator	Outcome	Method	Number.of.SNPs	Beta	Se	Pval	LO_CI	UP_CI	OR	OR_LCI95	OR_UCI95	Rucker Q(MR-Egger)	Pval_Q(MR-Egger)	Cochran Q(IVW)	Pval_Q(IVW)	Intercept(MR-Egger)	Pval_Intercept	Power	Steiger P-value	Number of SNP Not Passing MR-Steiger
Inflammatory bowel disease (IIBDGC)	Colorectal cancer (Huyghe JR et al.)	MR Egger	108	-0.037669021	0.062330897	0.546909328	-0.159837579	0.084499537	0.963031632	0.852282206	1.088172341	81.87799006	0.960414379	82.05510433	0.96506401	-0.00379059	0.67471754			
Inflammatory bowel disease (IIBDGC)	Colorectal cancer (Huyghe JR et al.)	Inverse variance weighted	108	-0.061564989	0.025712558	0.016649632	-0.111961603	-0.011168375	0.940291835	0.894078586	0.98889376	81.87799006	0.960414379	82.05510433	0.96506401	-0.00379059	0.67471754			
Inflammatory bowel disease (IIBDGC)	Colorectal cancer (Huyghe JR et al.)	Weighted median	108	-0.048720981	0.041308497	0.238222177	-0.129685635	0.032243674	0.952446844	0.878371517	1.032769133	81.87799006	0.960414379	82.05510433	0.96506401	-0.00379059	0.67471754	0.972401462356547	1.56918906830179e-128	0
Inflammatory bowel disease (IIBDGC)	Colorectal cancer (Huyghe JR et al.)	Weighted mode	108	-0.061228917	0.062569323	0.329996255	-0.183864791	0.061406956	0.940607894	0.832048297	1.063331556	81.87799006	0.960414379	82.05510433	0.96506401	-0.00379059	0.67471754			
Inflammatory bowel disease (IIBDGC)	Colorectal cancer (Huyghe JR et al.)	Simple mode	108	-0.153098401	0.094170901	0.106944545	-0.337673366	0.031476565	0.858045286	0.71342828	1.031977191	81.87799006	0.960414379	82.05510433	0.96506401	-0.00379059	0.67471754			

Mediator	Outcome	Method	Number.of.SNPs	Beta	Se	Pval	LO_CI	UP_CI	OR	OR_LCI95	OR_UCI95	Rucker Q(MR-Egger)	Pval_Q(MR-Egger)	Cochran Q(IVW)	Pval_Q(IVW)	Intercept(MR-Egger)	Pval_Intercept	Power	Steiger P-value	Number of SNP Not Passing MR-Steiger
Inflammatory bowel disease (Mbatchou J et al.)	Colorectal cancer (Ishigaki K et al)	MR Egger	33	40.04200337	16.36710957	0.020295391	7.962468606	72.12153814	2.45483E+17	2871.151953	2.1E+31	24.15463174	0.804184132	27.51197218	0.693271168	-0.034690447	0.076528349	0.53717903664665	1.11887369862568e-27	0
Inflammatory bowel	Colorectal cancer	Inverse	33	10.9579	3.9916	0.0060	3.13443	18.7815	57411.	22.9756	1434581	24.15463174	0.804184132	27.51197218	0.6932711	-0.034690447	0.0765283			

disease (Mbatchou J et al.)	(Ishigaki K et al.)	variance weighted		9397	1207	46385	4309	5362	15871	3505	08.5				68		49
Inflammatory bowel disease (Mbatchou J et al.)	Colorectal cancer (Ishigaki K et al.)	Weighted median	33	14.43296332	5.628661443	0.01034166	3.400786893	25.46513975	1854198.936	29.98768786	1.14649E+11	24.15463174	0.804184132	27.51197218	0.693271168	-0.034690447	0.076528349
Inflammatory bowel disease (Mbatchou J et al.)	Colorectal cancer (Ishigaki K et al.)	Weighted mode	33	15.73404964	9.001264046	0.090057281	-1.908427893	33.37652717	6811000.856	0.148313368	3.12782E+14	24.15463174	0.804184132	27.51197218	0.693271168	-0.034690447	0.076528349
Inflammatory bowel disease (Mbatchou J et al.)	Colorectal cancer (Ishigaki K et al.)	Simple mode	33	15.34475436	10.81960319	0.165787577	-5.861667902	36.55117662	4614685.883	0.002846492	7.48125E+15	24.15463174	0.804184132	27.51197218	0.693271168	-0.034690447	0.076528349

Abbreviation: beta, effect size; Se, Standard Error; Pval, P-value; CI, confidential interval; LO_CI, lower bound of the confidence interval; UP_CI, upper bound of the confidence interval; OR: odds ratio; OR_LCI95: lower 95% confidence interval for the odds ratio; OR_UCI95: upper 95% confidence interval for the odds ratio; P-adj, P-value after P-value adjustment

Table S5:

Detailed MR analyses between microbiota and colorectal cancer using Huyghe JR et al.'s GWAS data.

Trait	OR(IVW)	95%CI(IVW)	Pval(IVW)	OR(MR-Egger)	95%CI(MR-Egger)	Pval(MR-Egger)	OR(Weighted median)	95%CI(Weighted median)	Pval(Weighted median)	OR(Weighted mode)	95%CI(Weighted mode)	Pval(Weighted mode)	OR(Simple mode)	95%CI(Simple mode)	Pval(Simple mode)	Rucker Q(MR-Egger)	Pval_Q(MR-Egger)	Cochran Q(IVW)	Pval_Q (IVW)	Intercept(MR-Egger)	Pval_Intercept	Power	Steiger P-value	Number of SNP Not Passing MR-Steiger	P-adj(IVW)
class.Actinobacteria.id.419	0.842581591	0.810-0.876	8.02E-18	0.524617494	0.432-0.637	1.46E-10	0.835386014	0.794-0.879	4.38427E-12	0.782721879	0.658-0.931	5.76E-03	0.979043962	0.809-1.185	0.828118617	287.4558231	1	311.3955761	1	0.040261056	1.28187E-06	1	NA	0	1.880244E-16
family.Alcaligenaceae.id.2875	0.499851935	0.439-0.569	9.73156E-26	9.017912364	4.103-19.823	3.95626E-07	0.403955307	0.341-0.479	1.38995E-25	0.392245201	0.274-0.562	1.79277E-06	0.390193776	0.274-0.556	1.2313E-06	54.70517817	0.998787484	107.7361215	0.111141203	-0.169277471	1.19659E-10	0.963884339554322	2.7203292114425e-57	0	4.106060E-24
family.Bifidobacteriaceae.id.433	0.889010242	0.854-0.925	8.23E-09	1.101375913	0.913-1.329	3.14E-01	0.896440696	0.850-0.946	5.9974E-05	0.985080171	0.829-1.170	8.64E-01	0.985080171	0.824-1.178	0.869107937	152.7462636	1	157.9886142	1	-0.018560637	0.022461775	1	NA	0	5.988034E-08
genus..Eubacteriumcoprostanoligenesgroup.id.11375	1.610206236	1.358-1.910	4.37599E-08	5.874841011	1.708-20.206	7.26E-03	1.78370811	1.421-2.238	5.8333E-07	1.794219508	1.139-2.828	0.015226659	1.794219508	1.159-2.778	0.011745154	20.88439929	0.999470502	25.18377561	0.996188764	-0.100099334	0.043753185	0.74367451235922	3.72367542287884e-43	0	2.981226E-07
genus.Bifidobacterium.id.436	0.864013983	0.831-0.898	1.47E-13	1.099438845	0.910-1.328	3.26E-01	0.83991041	0.798-0.884	1.79251E-11	0.792466079	0.664-0.945	9.94E-03	0.979869497	0.809-1.187	0.835067449	156.442265	1	162.9564645	1	-0.020548542	0.010975272	1	NA	0	2.385923E-12
genus.Lachnoclostridium.id.11308	0.603759037	0.518-0.704	1.11685E-10	2.175147627	0.422-11.220	0.357002299	0.528384365	0.433-0.644	2.73448E-10	0.492071727	0.325-0.746	0.001438731	0.489691285	0.322-0.744	0.001427779	29.89783016	0.999428367	32.26294109	0.998722144	-0.069123229	0.129421273	0.833545111367607	3.13921574856696e-50	0	1.074182E-09
genus.LachnospiraceaeND3007group.id.11317	1.781045422	1.237-2.564	0.001905531	0.497274584	0.000-1438.000	0.867851505	1.835055662	1.189-2.833	0.006150304	1.834502968	1.013-3.323	0.076300914	1.834502968	0.962-3.498	0.098449733	0.087821306	0.99999985	0.186476508	0.999999591	0.070533909	0.761483895	0.238203846948318	1.29930151184086e-10	0	6.775551E-03
genus.Peptococcus.id.2037	0.924387539	0.882-0.969	0.000981388	0.567210125	0.416-0.773	4.10E-04	0.900912629	0.848-0.957	0.000776072	0.900019848	0.750-1.081	0.26006481	0.897993607	0.746-1.082	0.258193909	46.88843617	1	56.70145913	1	0.057405566	0.001998429	0.99999999999999997	0	0	3.907035E-03
genus.RuminococcaceaeUCG011.id.11368	1.370879786	1.314-1.430	6.39331E-49	0.185559769	0.125-0.276	1.89E-14	1.541999391	1.452-1.637	1.12745E-45	1.5460113	1.298-1.842	2.39685E-06	1.5460113	1.316-1.816	3.24875E-07	32.88733237	1	132.2282065	0.996443092	0.2469147	7.14474E-19	1	NA	0	4.494300E-47
genus.Streptococcus.id.1853	0.415155264	0.384-0.449	4.85E-109	0.217810745	0.156-0.305	7.97E-16	0.40082692	0.362-0.444	1.30E-67	0.415142424	0.314-0.548	3.85E-09	0.415142424	0.312-0.552	9.36706E-09	99.49314424	0.999998893	114.4187101	0.99988123	0.069640338	0.000157613	0.999963958280487	2.40946899567747e-162	0	1.023350E-106
genus.unknowngenus.id.1868	0.875060196	0.804-0.952	0.001913126	1.831098814	0.945-3.547	7.56E-02	1.026731587	0.919-1.147	0.641655519	1.036699774	0.770-1.395	0.812543463	1.036699774	0.766-1.403	0.816005776	50.66958322	0.999999956	55.54102398	0.999999462	-0.059098217	0.029308037	0.999698714462616	4.56291277433065e-244	0	6.775551E-03
order.Bifidobacteriales.id.432	0.889010242	0.854-0.925	8.23E-09	1.101375913	0.913-1.329	3.14E-01	0.896440696	0.850-0.946	6.1557E-05	0.985080171	0.822-1.180	8.71E-01	0.985080171	0.816-1.189	0.875402067	152.7462636	1	157.9886142	1	-0.018560637	0.022461775	1	NA	0	5.988034E-08
phylum.Actinobacteria.id.400	0.844439614	0.791-0.901	3.28699E-07	0.839356902	0.536-1.313	0.443920901	0.823376792	0.757-0.896	6.30241E-06	0.732993173	0.562-0.956	0.022538859	0.969760409	0.738-1.273	0.82533623	42.5489992	1	42.54971279	1	0.000420606	0.978707522	0.99999958723007	0	0	2.103606E-06

Abbreviation: OR, odds ratio; IVW, inverse variance weighted method; CI, confidential interval; a Pval of 0 means Pval<0.001; P-adj, P-value after P-value adjustment

Table S6:

Detailed MR analyses between microbiota and colorectal cancer using Ishigaki K et al.'s GWAS data.

Trait	OR(IVW)	95%CI(IVW)	Pval(IVW)	OR(MR-Egger)	95%CI(MR-Egger)	Pval(MR-Egger)	OR(Weighted median)	95%CI(Weighted median)	Pval(Weighted median)	OR(Weighted mode)	95%CI(Weighted mode)	Pval(Weighted mode)	OR(Simple mode)	95%CI(Simple mode)	Pval(Simple mode)	Rucker Q(MR-Egger)	Pval_Q(MR-Egger)	Cochran Q(IVW)	Pval_Q (IVW)	Intercept(MR-Egger)	Pval_Intercept	Power	Steiger P-value	Number of SNP Not Passing MR-Steiger	P-adj(IVW)
class.Actinobacteria.id.419	0.902549378	0.876-0.930	9.83922E-12	1.07762379	0.912-1.273	0.38067589	0.946322204	0.913-0.981	0.002508774	0.930875644	0.847-1.023	0.135701783	0.930875644	0.839-1.032	0.175900877	751.119065	2.73E-12	757.8183456	1.06676E-12	-0.015414768	0.035019823	1	NA	0	2.306933E-10
family.Alcaligenaceae.id.2875	1.507698592	1.389-1.636	8.3167E-23	0.376914677	0.213-0.667	0.0011993	1.677632117	1.499-1.877	1.67329E-19	1.711606538	1.339-2.187	4.37733E-05	1.717604257	1.344-2.195	3.92429E-05	34.62168962	0.999999965	57.70576616	0.996781624	0.079847493	6.24442E-06	0.999815215642094	1.00028142758826e-237	0	4.388800E-21
family.Bifidobacteriaceae.id.433	0.913761567	0.891-0.937	1.79224E-12	1.325693074	1.156-1.520	6.12702E-05	0.949959821	0.919-0.982	0.002700189	0.939361005	0.860-1.026	0.164844567	0.924457269	0.830-1.029	0.152353309	479.0092845	0.282744195	509.534164	0.066481811	-0.034424041	9.32049E-08	1	NA	0	4.721125E-11
genus..Eubacteriumcoprostanoligenesgroup.id.11375	1.221783912	1.118-1.336	1.06296E-05	0.771490391	0.314-1.893	0.57427869	1.371389214	1.217-1.545	2.17193E-07	1.403700167	1.109-1.777	0.007344196	1.403700167	1.095-1.800	0.010725136	20.94367078	0.994368355	21.96121767	0.993498	0.035646034	0.31916497	0.950653477896198	5.97187785645653e-112	0	7.712414E-05
genus.Bifidobacterium.id.436	0.88797759	0.865-0.912	1.46962E-18	1.30871109	1.128-1.519	0.00043527	0.946477028	0.912-0.982	0.003463361	0.926344743	0.847-1.013	0.094783079	0.911215674	0.826-1.006	0.064962308	604.7317853	1.63E-03	636.8425484	7.40558E-05	-0.034797968	3.15343E-07	1	NA	0	6.203400E-17
genus.Lachnoclostridium.id.11308	0.808627052	0.725-0.901	0.000126025	2.052019352	0.652-6.460	0.224303728	0.79998784	0.697-0.918	0.001482784	0.823209994	0.618-1.097	0.189838981	0.815145246	0.607-1.094	0.178940665	9.231355808	1	11.78728115	1	-0.050062946	0.115410598	0.988992378782742	8.86915171742314e-169	0	7.186831E-04
genus.LachnospiraceaeND3007group.id.11317	2.368381662	1.846-3.038	1.14099E-11	0.000748132	0.000-30.915	0.221040983	2.431709716	1.734-3.410	2.56481E-07	2.432209797	1.588-3.725	0.002732675	2.432209797	1.619-3.655	0.002055177	4.050961189	0.852496523	6.261190883	0.713519294	0.440396839	0.17540916	0.451543442352209	9.54909078153761e-17	0	2.405400E-10
genus.Peptococcus.id.2037	1.261420361	1.224-1.300	1.36808E-52	0.87010221	0.645-1.174	0.364176283	1.261631199	1.212-1.314	1.23842E-29	1.262213957	1.143-1.394	8.2026E-06	1.262213957	1.143-1.394	8.42771E-06	40.60641923	1.00E+00	46.56154201	1	0.042961311	0.015654364	1	0	0	2.890700E-50
genus.RuminococcaceaeUCG011.id.11368	1.249041803	1.203-1.297	2.74577E-31	1.631085751	1.238-2.148	0.000629966	1.235581931	1.174-1.300	3.73569E-16	1.220782986	1.073-1.389	0.002835237	1.220782986	1.064-1.401	0.005007309	13.85310275	1	17.52560487	1	-0.034266319	0.056928334	1	NA	0	2.901250E-29
genus.Streptococcus.id.1853	0.845166204	0.745-0.959	0.00914564	0.536098971	0.326-0.880	0.022051524	0.774681121	0.655-0.917	0.002934932	0.765612471	0.647-0.906	0.004846802	0.757939935	0.603-0.952	0.025987427	14.77300201	0.871794027	18.23370033	0.744692355	0.038944464	0.076265212	0.79804080627003	2.12387204981615e-59	0	2.837838E-02
genus.unknowngenus.id.1868	1.082271906	1.027-1.141	0.003144005	3.741654142	1.610-8.698	0.002736902	1.155054915	1.077-1.239	5.91343E-05	1.178110394	0.980-1.417	0.084575251	1.178110394	0.980-1.416	0.083953545	35.98947608	1	44.32852591	0.999999997	-0.0970788	0.004680574	0.999999995849101	0	0	1.184616E-02
order.Bifidobacteriales.id.432	0.913761567	0.891-0.937	1.79224E-12	1.325693074	1.156-1.520	6.12702E-05	0.949959821	0.918-0.983	0.003080105	0.939361005	0.859-1.027	0.169533148	0.924457269	0.834-1.025	0.136669591	479.0092845	0.282744195	509.534164	0.066481811	-0.034424041	9.32049E-08	1	NA	0	4.721125E-11
phylum.Actinobacteria.id.400	0.91271917	0.879-0.948	2.56415E-06	1.778464868	1.365-2.318	2.86942E-05	0.889985361	0.845-0.938	1.2194E-05	0.912469529	0.760-1.095	0.326082091	0.897902415	0.751-1.073	0.237398868	159.9795347	0.999999397	184.8629822	0.999733533	-0.047808697	1.12768E-06	0.99999999999998	0	0	2.250667E-05

Abbreviation: OR, odds ratio; IVW, inverse variance weighted method; CI, confidential interval; a Pval of 0 means Pval<0.001; P-adj, P-value after P-value adjustment

Table S7:

The Mendelian randomization results of the microbiota and IBD which had mediating effects in CRC.

Id.exposure	Exposure	Id.outcome	Outcome	Method	Nsnp	β	Se	Pval	LO_CI	UP_CI	OR	OR_LCI95	or_UCI95
MiBioGen	family.Alcaligenaceae.id.2875	ebi-a-GCST012880	Colorectal cancer (Huyghe JR et al.)	Inverse variance weighted	92	-0.693443355	0.066113111	9.73156E-26	-0.823025054	-0.563861657	0.499851935	0.439101338	0.569007504
MiBioGen	family.Alcaligenaceae.id.2875	finn-b-K11_IBD	Inflammatory bowel disease (FinnGen)	Inverse variance weighted	91	0.246063224	0.039125338	3.19E-10	0.169377561	0.322748887	1.278980433	1.184567301	1.380918541
finn-b-K11_IBD	Inflammatory bowel disease (FinnGen)	ebi-a-GCST012880	Colorectal cancer (Huyghe JR et al.)	Inverse variance weighted	37	-0.10710114	0.048820055	0.028250229	-0.202788448	-0.011413832	0.898434801	0.816450945	0.988651059
MiBioGen	genus.Streptococcus.id.1853	ebi-a-GCST012880	Colorectal cancer (Huyghe JR et al.)	Inverse variance weighted	176	-0.879102699	0.039626881	4.847457298E-109	-0.956771384	-0.801434013	0.415155264	0.384131098	0.448685082
MiBioGen	genus.Streptococcus.id.1853	ieu-a-31	Inflammatory bowel disease (IIBDGC)	Inverse variance weighted	173	0.348394223	0.021400544	1.375635820E-59	0.306449158	0.390339289	1.416790672	1.358592392	1.477482003
MiBioGen	genus.unknowngenus.id.1868	ebi-a-GCST012880	Colorectal cancer (Huyghe JR et al.)	Inverse variance weighted	116	-0.1334626	0.043005322	0.001913126	-0.217753032	-0.049172168	0.875060196	0.80432406	0.952017209
MiBioGen	genus.unknowngenus.id.1868	ieu-a-31	Inflammatory bowel disease (IIBDGC)	Inverse variance weighted	115	0.066813525	0.023090286	0.003808806	0.021556565	0.112070485	1.069096100	1.021790587	1.118591701
MiBioGen	genus.Peptococcus.id.2037	ebi-a-GCST012880	Colorectal cancer (Huyghe JR et al.)	Inverse variance weighted	198	-0.07862388	0.023855717	0.000981388	-0.125381086	-0.031866675	0.924387539	0.882160659	0.968635717
MiBioGen	genus.Peptococcus.id.2037	ieu-a-31	Inflammatory bowel disease (IIBDGC)	Inverse variance weighted	195	0.153335427	0.012717996	1.790845880E-33	0.128408154	0.178262700	1.165715926	1.137016986	1.195139243
ieu-a-31	Inflammatory bowel disease (IIBDGC)	ebi-a-GCST012880	Colorectal cancer (Huyghe JR et al.)	Inverse variance weighted	108	-0.061564989	0.025712558	0.016649632	-0.111961603	-0.011168375	0.940291835	0.894078586	0.98889376
MiBioGen	class.Actinobacteria.id.419	bbj-a-76	Colorectal Cancer (Ishigaki K et al.)	Inverse variance weighted	503	-0.102531878	0.015058652	9.83922E-12	-0.132046836	-0.07301692	0.902549378	0.876299952	0.929585102
MiBioGen	class.Actinobacteria.id.419	ebi-a-GCST90038683	Inflammatory bowel disease (Mbatchou J et al.)	Inverse variance weighted	599	-0.003461744	0.000150239	1.7847E-117	-0.003756211	-0.003167276	0.996544241	0.996250834	0.996837735
MiBioGen	family.Bifidobacteriaceae.id.433	bbj-a-76	Colorectal Cancer (Ishigaki K et al.)	Inverse variance weighted	464	-0.090185609	0.012792717	1.79224E-12	-0.115259334	-0.065111884	0.913761567	0.891135012	0.936962626
MiBioGen	family.Bifidobacteriaceae.id.433	ebi-a-GCST90038683	Inflammatory bowel disease (Mbatchou J et al.)	Inverse variance weighted	502	-0.00375932	0.000141424	1.097E-155	-0.00403651	-0.00348213	0.996247737	0.995971625	0.996523926
MiBioGen	genus.Eubacteriumcoprostanoligen esgroup.id.11375	bbj-a-76	Colorectal Cancer (Ishigaki K et al.)	Inverse variance weighted	42	0.200312014	0.045484592	1.06296E-05	0.111162214	0.289461814	1.221783912	1.117576178	1.335708434
MiBioGen	genus.Eubacteriumcoprostanoligen esgroup.id.11375	ebi-a-GCST90038683	Inflammatory bowel disease (Mbatchou J et al.)	Inverse variance weighted	48	0.005821893	0.000552299	5.57818E-26	0.004739387	0.006904398	1.005838873	1.004750636	1.006928289
MiBioGen	genus.Bifidobacterium.id.436	bbj-a-76	Colorectal Cancer (Ishigaki K et al.)	Inverse variance weighted	508	-0.118808772	0.01351333	1.46962E-18	-0.145294899	-0.092322646	0.88797759	0.864767237	0.91181091
MiBioGen	genus.Bifidobacterium.id.436	ebi-a-GCST90038683	Inflammatory bowel disease (Mbatchou J et al.)	Inverse variance weighted	547	-0.003345587	0.000150093	4.6159E-110	-0.00363977	-0.003051404	0.996660004	0.996366846	0.996953247
MiBioGen	genus.RuminococcaceaeUCG011.id. 11368	bbj-a-76	Colorectal Cancer (Ishigaki K et al.)	Inverse variance weighted	179	0.2223767	0.019113242	2.74577E-31	0.184914745	0.259838654	1.249041803	1.203115864	1.296720849
MiBioGen	genus.RuminococcaceaeUCG011.id. 11368	ebi-a-GCST90038683	Inflammatory bowel disease (Mbatchou J et al.)	Inverse variance weighted	180	0.001243861	0.000127722	2.06004E-22	0.000993525	0.001494197	1.001244635	1.000994019	1.001495314

MiBioGen	genus.unknowngenus.id.1868	bbj-a-76	Colorectal Cancer (Ishigaki K et al.)	Inverse variance weighted	111	0.079062448	0.02677092	0.003144005	0.026591444	0.131533452	1.082271906	1.026948152	1.140576061
MiBioGen	genus.unknowngenus.id.1868	ebi-a-GCST90038683	Inflammatory bowel disease (Mbatchou J et al.)	Inverse variance weighted	116	0.000738217	0.000256157	0.003952954	0.000236149	0.001240285	1.000738489	1.000236177	1.001241054
MiBioGen	order.Bifidobacteriales.id.432	bbj-a-76	Colorectal Cancer (Ishigaki K et al.)	Inverse variance weighted	464	-0.090185609	0.012792717	1.79224E-12	-0.115259334	-0.065111884	0.913761567	0.891135012	0.936962626
MiBioGen	order.Bifidobacteriales.id.432	ebi-a-GCST90038683	Inflammatory bowel disease (Mbatchou J et al.)	Inverse variance weighted	502	-0.00375932	0.000141424	1.097E-155	-0.00403651	-0.00348213	0.996247737	0.995971625	0.996523926
MiBioGen	phylum.Actinobacteria.id.400	bbj-a-76	Colorectal Cancer (Ishigaki K et al.)	Inverse variance weighted	257	-0.091327036	0.019419049	2.56415E-06	-0.129388372	-0.0532657	0.91271917	0.878632663	0.948128061
MiBioGen	phylum.Actinobacteria.id.400	ebi-a-GCST90038683	Inflammatory bowel disease (Mbatchou J et al.)	Inverse variance weighted	283	-0.004644301	0.00024121	1.30257E-82	-0.005117073	-0.004171529	0.995366467	0.994895997	0.99583716
ebi-a-GCST90038683	Inflammatory bowel disease (Mbatchou J et al.)	bbj-a-76	Colorectal Cancer (Ishigaki K et al.)	Inverse variance weighted	33	10.95799397	3.99161207	0.006046385	3.134434309	18.78155362	57411.15871	22.97563505	143458108.5

Abbreviation: β, effect size; Se, Standard Error; Pval, P-value; CI, confidential interval; LO_CI, lower bound of the confidence interval; UP_CI, upper bound of the confidence interval; OR: odds ratio; OR_LCI95: lower 95% confidence interval for the odds ratio; OR_UCI95: upper 95% confidence interval for the odds ratio; P-adj, P-value after P-value adjustment

Table S8:

Expression analysis and LASSO regression.

Gene	P-val	P-adj	LASSO Coefficient
ADCYAP1R1	2.031873365E-28	3.11E-27	-0.72041028
AHRR	0.008265042	0.010560887	.
ARHGAP26	2.678206030E-12	6.85E-12	.
ATP11A	6.315772822E-25	4.15E-24	-0.500913326
AXDND1	1.331285748E-15	5.10E-15	.
BCAS3	9.358943149E-10	2.15E-09	.
BVES	1.036509562E-13	3.19E-13	.
DEPDC4	3.644700496E-26	2.79E-25	.
DOCK2	2.855396332E-08	5.98E-08	.
DOCK4	5.132016487E-15	1.69E-14	.
FAM120A	6.172990050E-27	5.68E-26	-0.666156045
GAS2L3	6.027631421E-10	1.46E-09	.
GBE1	6.726785945E-32	3.10E-30	-0.925015451
KIF14	9.621553077E-08	1.77E-07	.
LCT	0.000795597	0.001180563	.
LIN28B	0.001470550	0.001989568	.
MAP3K13	4.009357076E-13	1.15E-12	.
MBLAC2	0.000327083	0.000518821	.
MCM6	1.210432791E-29	2.78E-28	-0.473108172
MEIS2	8.862220358E-07	1.57E-06	.
MGA	2.865189635E-08	5.74E-08	.
MSRA	4.259842744E-20	1.96E-19	0.754643932
NR1H4	8.899476455E-05	0.000146214	.
PMS1	1.526567843E-15	5.41E-15	.
RPTOR	1.2727603145E-27	1.46E-26	.
SLC17A8	0.001101233	0.001535052	.
SLC9A3	3.889350112E-08	7.46E-08	.
SOAT1	2.952278631E-24	1.70E-23	0.226702616
ST8SIA2	0.000792786	0.001215605	.
STIM1	0.000828942	0.001191604	.
TBC1D5	4.745091713E-06	8.09E-06	.
THSD7B	0.001924538	0.002529393	.
TMTC1	1.235057525E-08	2.72E-08	.
UBXN4	1.064349637E-18	4.43E-18	.
WAC	2.315678573E-12	6.28E-12	.
ZDHHC4	2.594442230E-23	1.32E-22	0.02477055
ZRANB3	0.019642490	0.024420393	.

Abbreviation: LASSO, least absolute shrinkage and selection operator; P-val, P-value; P-adj, P-value after P-value adjustment

Table S9:

Detailed results of drug target MR analyses between seven candidate genes and colorectal cancer using Huyghe JR et al.'s GWAS data.

Gene	OR(IVW)	95%CI(IVW)	Pval(IVW)	OR(MR-Egger)	95%CI(MR-Egger)	Pval(MR-Egger)	OR(Weighted median)	95%CI(Weighted median)	Pval(Weighted median)	OR(Weighted mode)	95%CI(Weighted mode)	Pval(Weighted mode)	OR(Simple mode)	95%CI(Simple mode)	Pval(Simple mode)	Rucker Q(MR-Egger)	Pval_Q(MR-Egger)	Cochran Q(IVW)	Pval_Q(IVW)	Intercept(MR-Egger)	Pval_Intercept	Power	Steiger P-value	Number of SNP Not Passing MR-Steiger	P-adj(IVW)
ADCYAP1R1	N/A																								
ATP11A	1.361190184	1.173-1.580	5.06936E-05	1.028230705	0.540-1.958	0.932754316	1.441496654	1.179-1.763	0.000372243	1.632707759	1.080-2.467	0.022893531	1.641462443	1.014-2.658	0.047805364	24.62137339	0.999999726	25.3908879	0.999999676	0.016497806	0.383456013	0.862119388167661	2.0696151513488e-76	0	5.069360E-05
FAM120A	0.752199959	0.716-0.791	5.26086E-29	1.06385071	0.913-1.239	0.427114844	0.727164415	0.679-0.778	4.32525E-20	0.754229789	0.632-0.901	0.001981141	0.799549964	0.649-0.984	0.035657347	147.8554711	1	170.0567747	1	-0.025196653	3.45551E-06	0.999999999999881	0	0	1.227534E-28
GBE1	1.08211322	1.052-1.113	5.58027E-08	0.767236352	0.705-0.835	1.59645E-09	1.071122207	1.029-1.115	0.00087391	1.054871904	0.975-1.142	0.186437057	1.064604488	0.963-1.177	0.222452882	694.6686905	0.93974023	765.3789555	0.388581463	0.034515593	2.06047E-16	1	NA	2	6.510315E-08
MCM6	0.968912025	0.963-0.975	7.90248E-23	0.989283338	0.978-1.001	0.075139988	0.958223515	0.950-0.967	3.4972E-20	0.958343078	0.939-0.978	2.74846E-05	0.958343078	0.934-0.984	0.001315725	1786.909287	1	1803.357056	1	-0.00655054	5.12395E-05	1	NA	0	1.382934E-22
MSRA	0.941217558	0.935-0.947	1.62454E-74	0.99029247	0.973-1.007	0.26533864	0.940962413	0.932-0.950	7.64282E-35	0.913885368	0.888-0.940	6.57851E-10	0.966345123	0.929-1.005	0.087293325	3959.601457	1	3998.932162	1	-0.008874538	3.82999E-10	1	NA	1	1.137178E-73
SOAT1	1.071722014	1.063-1.081	1.88516E-57	1.127084491	1.111-1.143	1.43426E-59	1.067621907	1.056-1.079	7.281E-32	1.09501205	1.072-1.119	1.48149E-16	1.061398181	1.033-1.091	1.89579E-05	3738.638878	2.44415E-32	3841.950157	2.32643E-38	-0.013473517	3.77085E-18	1	NA	7	6.598060E-57
ZDHHC4	0.94985642	0.939-0.961	9.09608E-18	0.924521842	0.907-0.942	4.23124E-16	0.943841502	0.928-0.960	1.79043E-11	0.945591717	0.911-0.982	0.003393716	0.945591717	0.900-0.994	0.026884641	1279.138727	1	1292.254011	1	0.006637313	0.000299956	1	NA	0	1.273451E-17

Abbreviation: OR, odds ratio; IVW, inverse variance weighted method; CI, confidential interval; a Pval of 0 means P-value <0.001; P-adj, P-value after P-value adjustment

Table S10:

Detailed results of drug target MR analyses between seven candidate genes and colorectal cancer using Ishigaki K et al.’s GWAS data.

Gene	OR(IVW)	95%CI(IVW)	Pval(IVW)	OR(MR-Egger)	95%CI(MR-Egger)	Pval(MR-Egger)	OR(Weighted median)	95%CI(Weighted median)	Pval(Weighted median)	OR(Weighted mode)	95%CI(Weighted mode)	Pval(Weighted mode)	OR(Simple mode)	95%CI(Simple mode)	Pval(Simple mode)	Rucker Q(MR-Egger)	Pval_Q(MR-Egger)	Cochran Q(IVW)	Pval_Q(IVW)	Intercept(MR-Egger)	Pval_Intercept	Power	Steiger P-value	Number of SNP Not Passing MR-Steiger	P-adj(IVW)
ADCYAP1R1													N/A												
ATP11A	0.944597882	0.855-1.044	0.262956003	0.936827512056681	0.595-1.474	0.778717961604112	1.02893739312216	0.890-1.189	0.699388873076929	1.18394644808038	0.833-1.682	0.349349941722174	1.17368790645849	0.815-1.691	0.39317826172341	47.6193835292017	0.937432933999965	47.6207243023931	0.948059860580303	0.000490205746277876	0.970904704571206	0.992659650897038	2.3879048401672 5E-205	0	3.067820E-01
FAM120A	0.840863471	0.813-0.869	1.018229135E-24	0.973028681378124	0.852-1.112	0.687814510920196	0.907346524593624	0.866-0.951	4.31639885897264E-05	0.912062142151456	0.831-1.001	0.0522522679805121	0.936796029836376	0.831-1.056	0.28482256767996	270.694431383211	0.991728750495853	275.608610087265	0.986784584040034	-0.010029436	0.0273205186095005	1	0	0	2.375868E-24
GBE1	0.928667517805	0.912-0.946	1.17202424477E-15	0.935705868211616	0.884-0.990	0.0209580031918368	0.913420422097156	0.888-0.939	1.80182135350587E-10	0.847976503757413	0.792-0.908	3.17595952411839E-06	0.997252542714367	0.913-1.089	0.951099252364387	523.639242823224	0.99999999899324	523.716333733508	0.999999999137615	-0.000793695	0.781357740058493	1	NA	0	2.051042E-15
MCM6	0.968743043	0.965-0.972	7.487092450E-64	0.969914788001067	0.963-0.977	1.38144378494444E-16	0.966005235960148	0.962-0.970	5.32471300644235E-50	0.970048295411662	0.957-0.984	2.06555242053737E-05	0.970048295411662	0.956-0.984	2.34436382439955E-05	3345.77219241229	5.46885026756827E-19	3345.95785138174	6.03414085089972E-19	-0.000411209	0.701347565002985	1	NA	0	5.240965E-63
MSRA	1.029855315	1.025-1.035	2.105727205E-29	1.05371297358826	1.039-1.069	1.59074552177676E-12	1.0326426296063	1.024-1.041	2.24402676276855E-15	1.04000482603289	1.002-1.079	0.0364152028330431	0.999107315159646	0.957-1.043	0.967819901163503	3850.55407115046	0.999999919138341	3861.55643957649	0.999999848781547	-0.003712278	0.000917477646177506	1	NA	0	7.370045E-29
SOAT1	1.005761443	0.997-1.014	0.179558459	1.02076273568425	1.005-1.037	0.00942505046027771	0.974610791171137	0.964-0.985	1.45189913229637E-06	0.924672641794078	0.900-0.950	1.09681770071163E-08	1.08996460088761	1.050-1.132	6.98578614515246E-06	3438.20376747688	2.90087019200454E-58	3445.95536805253	8.89430509020962E-59	-0.002906296	0.0261428740835052	1	NA	0	2.513818E-01
ZDHHC4	1.002668506	0.992-1.014	0.632127021	0.950801579640441	0.933-0.969	1.44072787724616E-07	0.985551495895111	0.970-1.001	0.0748701735430679	1.38778785993668	0.137-14.041	0.781405509707096	1.38778785993668	0.127-15.158	0.788233606746442	1508.65037394629	6.98132805303728E-05	1562.08590682162	1.12782625085564E-06	0.0105997573791103	1.60204462280599E-11	1	NA	0	6.321270E-01

Abbreviation: OR, odds ratio; IVW, inverse variance weighted method; CI, confidential interval; a Pval of 0 means P-value <0.001; P-adj, P-value after P-value adjustment

Table S11:
Detailed results of SMR and HEIDI tests between the expression of seven candidate genes and colorectal cancer using Ishigaki K et al.'s GWAS data.

Gene	ProbeID	ProbeChr	Probe_bp	topSNP	topSNP_chr	topSNP_bp	A1	A2	Freq	Beta_GWAS	Se_GWAS	P_GWAS	Beta_eQTL	Se_eQTL	P_eQTL	Beta_SMR	Se_SMR	P_SMR	P_HEIDI	Nsnp_HEIDI	OR	LO_CI	UP_CI
ADCYAP1R1													N/A										
ATP11A	ENSG00000068650	13	113443062	rs7998551	13	113540649	0	G	0.214712	-0.02	0.026	0.4463998	-0.0931302	0.0116186	1.095995e-15	0.214753	0.280462	0.4438472	0.5920688	18	1.23955568889576	0.715372051411224	2.14783105216759
*FAM120A	ENSG00000165233	9	95867032	rs10821082	9	95943304	C	G	0.385686	0.04	0.021	0.05573012	0.160459	0.0120975	3.75377E-40	0.249286	0.132218	0.05937317	0.2275653	20	1.28310894975785	0.990187182988429	1.6626841926795
GBE1	ENSG00000114480	3	81675081	rs11715625	3	81875938	T	A	0.331014	0.008	2.00E-02	0.6906993	-0.155264	0.00824703	4.571817e-79	-0.0515253	0.128842	0.689223	0.06243605	20	0.949779620233833	0.737819827770496	1.22263090941509
*MCM6	ENSG00000231890	2	136761317	rs2278682	2.00E+00	136743143	C	G	0.209742	-0.045	0.021	0.03522979	-0.208136	0.013788	1.74E-51	0.216205	0.101907	0.03387238	0.1146984	20	1.24135683106881	1.01660361297165	1.51579904141476
*MSRA	ENSG00000079459	8	11674950	rs6601572	8.00E+00	11093337	C	G	0.417495	-0.04	2.00E-02	0.04537013	0.239012	8.71E-03	8.5615E-166	-1.67E-01	0.0838999	4.61E-02	0.2590751	20	0.845898417891167	0.717630975325375	0.997092040329433
SOAT1	ENSG00000186283	1	179058835	rs1128952	1	179065125	T	C	0.2833	-0.035	2.20E-02	0.1191999	-0.218185	1.27E-02	7.646896e-66	1.60E-01	0.101265	1.13E-01	0.5002422	20	1.17399680507382	0.962649981523659	1.43174416950804
*ZDHHC4	ENSG00000164535	7	6486289	rs13235365	7.00E+00	6456091	T	C	0.238569	-0.075	0.027	0.006440951	0.625471	0.00872303	0	-1.20E-01	0.0431998	5.51E-03	0.250002	2.00E+01	0.887000263148598	0.814988229644134	0.965375251087032

Abbreviation: ProbeID, Probe Identifier; ProbeChr, Chromosome on which the probe is located; A1, Effect Allele; A2, Non-effect Allele; Freq, Frequency of the Effect Allele (A1); Se, Standard Error; P_SMR, P-value from Summary data-based Mendelian Randomization analysis; P_HEIDI, P-value from Heterogeneity in Dependent Instruments test; OR: odds ratio; LO_CI, lower bound of the confidence interval; UP_CI, upper bound of the confidence interval. * means that its corresponding gene pass the SMR (p_SMR < 0.1) and HEIDI test (p_HEIDI > 0.05).

Figure S1:

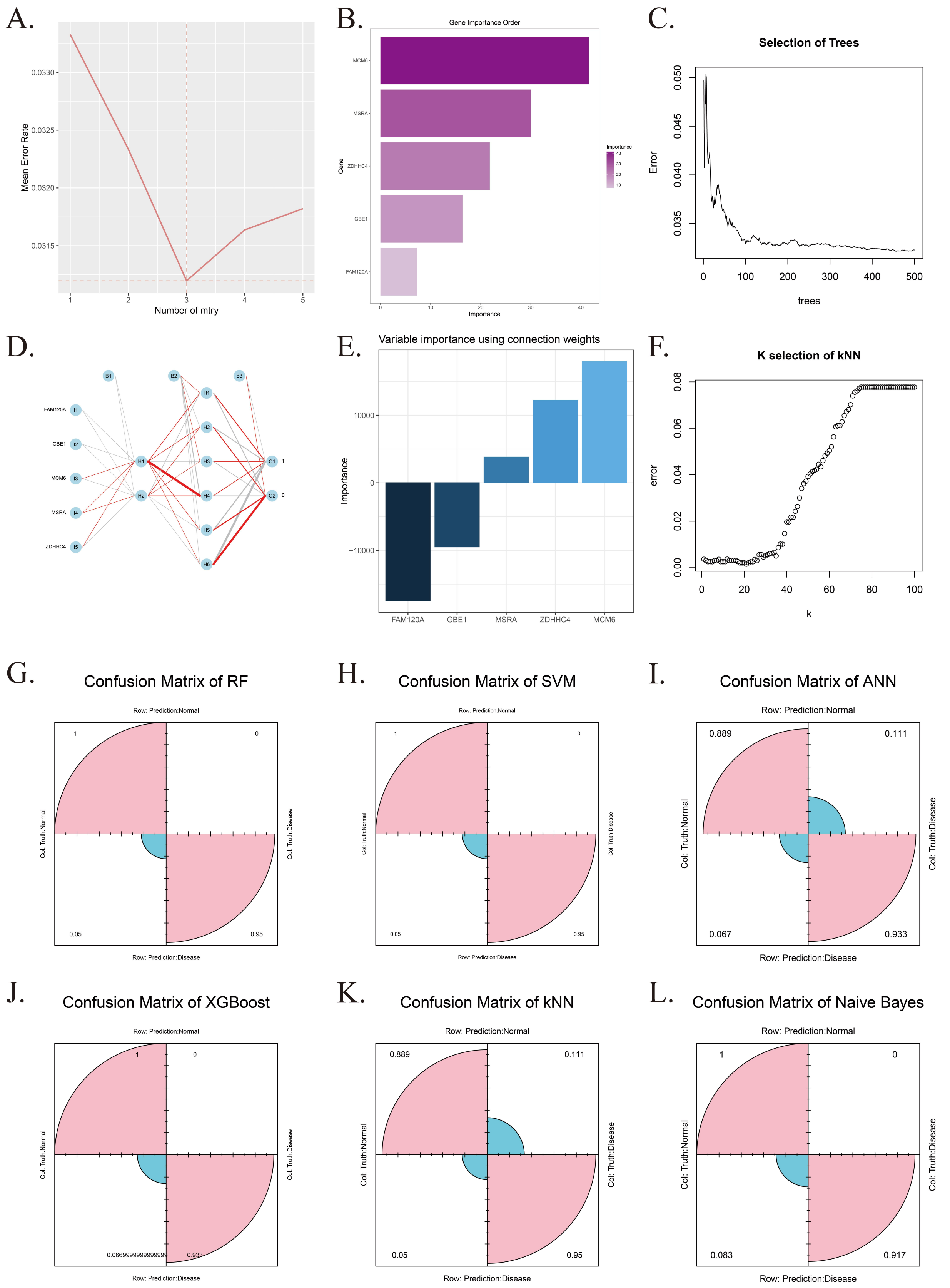

A.
Mean Error Rate
Number of mtry
B.
Gene Importance Order
C.
Selection of Trees
Error
trees
D.
E.
Variable importance using connection weights
Importance
FAM120A
GBE1
MSRA
ZDHHC4
MCM6
F.
K selection of kNN
error
k
G.
Confusion Matrix of RF
H.
Confusion Matrix of SVM
I.
Confusion Matrix of ANN
Row: Prediction:Normal
Col: Truth:Normal
Col: Truth:Disease
Row: Prediction:Disease
0.889
0.111
0.067
0.933
J.
Confusion Matrix of XGBoost
K.
Confusion Matrix of kNN
Row: Prediction:Normal
Col: Truth:Normal
Col: Truth:Disease
Row: Prediction:Disease
0.889
0.111
0.05
0.95
L.
Confusion Matrix of Naive Bayes
Row: Prediction:Normal
Col: Truth:Normal
Col: Truth:Disease
Row: Prediction:Disease
1
0
0.083
0.917

Parameter tuning, variable importance, and model performance evaluation of six machine learning algorithms used for CRC classification.

(A) Selection of the mtry parameter in the random forest (RF) model. The lowest mean error rate occurred at mtry = 3, indicating this as the optimal number of variables randomly sampled at each tree node split. (B) Variable importance in the RF model. MCM6 showed the highest predictive importance, followed by ZGHH4, MRSA, GBE1, and FAM120A, highlighting their contributions to CRC classification. (C) Selection of the number of trees (ntree) in the RF model. The classification error rate began to plateau around 500 trees, indicating model convergence. The final model was trained with ntree = 500 to ensure stability and prevent underfitting. (D) Schematic diagram of the artificial neural network (ANN) model, illustrating the connections between input gene features and output classes. Red and blue lines represent positive and negative connection weights, respectively, with line thickness reflecting the magnitude of influence. (E) Variable importance in the ANN model based on connection weights. MCM6 and ZDHHC4 contributed most positively to CRC prediction, whereas FAM120A and GBE1 showed negative associations. (F) Optimization of the k parameter in the k-nearest neighbors (kNN) model. The lowest error rate was achieved when k = 19. (G–L) Confusion matrix of the six machine learning models: RF, support vector machine (SVM), ANN, XGBoost, kNN, and Naive Bayes. Each matrix shows the distribution of true positives, true negatives, false positives, and false negatives. All models demonstrated high classification accuracy, particularly in discriminating CRC from normal samples.
